# Genomics and Transcriptomics of the green mussel explain the durability of its byssus

**DOI:** 10.1038/s41598-021-84948-6

**Published:** 2021-03-16

**Authors:** Koji Inoue, Yuki Yoshioka, Hiroyuki Tanaka, Azusa Kinjo, Mieko Sassa, Ikuo Ueda, Chuya Shinzato, Atsushi Toyoda, Takehiko Itoh

**Affiliations:** 1 Atmosphere and Ocean Research Institute, The University of Tokyo, Kashiwa, 277-8564 Japan; 2 Graduate School of Frontier Sciences, The University of Tokyo, Kashiwa, 277-8563 Japan; 3 Department of Biological Information, Tokyo Institute of Technology, Meguro, Tokyo, 152-8550 Japan; 4 School of Marine Biosciences, Kitasato University, Kanagawa, 252-0373 Japan; 5 Faculty of Science, Kanagawa University, Hiratsuka, 259-1293 Japan; 6 Comparative Genomics Laboratory, National Institute of Genetics, Mishima, 411-8540 Japan

**Keywords:** Physiology, Biochemistry

## Abstract

Mussels, which occupy important positions in marine ecosystems, attach tightly to underwater substrates using a proteinaceous holdfast known as the byssus, which is tough, durable, and resistant to enzymatic degradation. Although various byssal proteins have been identified, the mechanisms by which it achieves such durability are unknown. Here we report comprehensive identification of genes involved in byssus formation through whole-genome and foot-specific transcriptomic analyses of the green mussel, *Perna viridis*. Interestingly, proteins encoded by highly expressed genes include proteinase inhibitors and defense proteins, including lysozyme and lectins, in addition to structural proteins and protein modification enzymes that probably catalyze polymerization and insolubilization. This assemblage of structural and protective molecules constitutes a multi-pronged strategy to render the byssus highly resistant to environmental insults.

## Introduction

**Figure 1:**
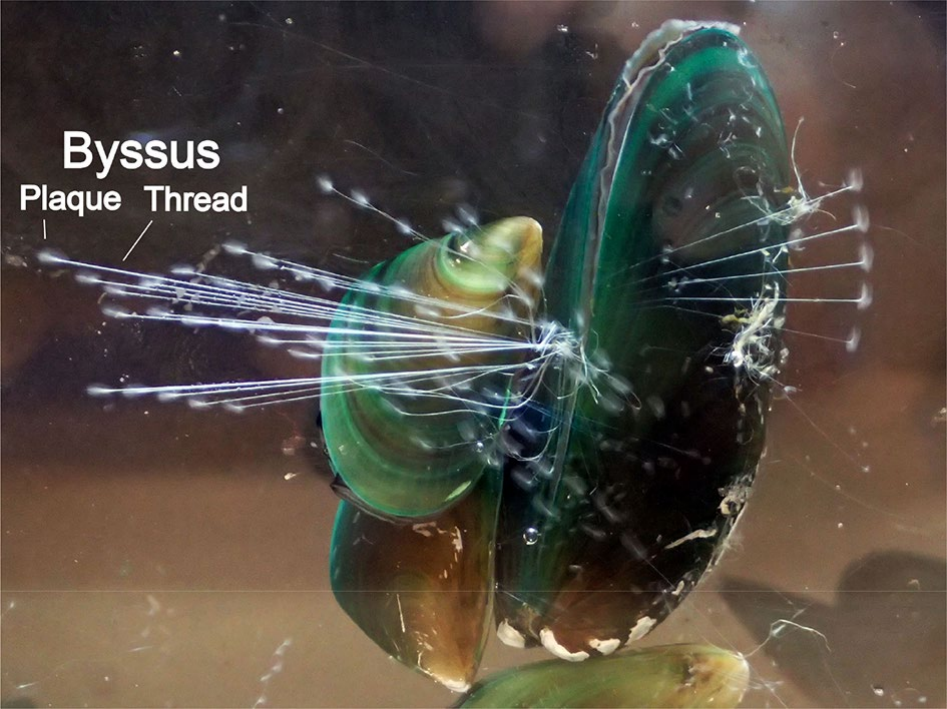
Green mussels, *Perna viridis*, attached to a transparent acrylic board, using the byssus, consisting of threads and plaques.

Mussels of the bivalve family Mytilidae occur in a variety of environments from freshwater to deep-sea. The family incudes ecologically important taxa such as coastal species of the genera *Mytilus* and *Perna*, the freshwater mussel, *Limnoperna fortuneri*, and deep-sea species of the genus *Bathymodiolus*, which constitute keystone species in their respective ecosystems^1^. One of the most important characteristics of mussels is their capacity to attach to underwater substrates using a structure known as the byssus, a proteinous holdfast consisting of threads and adhesive plaques (Fig. 1)^2^. Using the byssus, mussels often form dense clusters called “mussel beds.” The piled-up structure of mussel beds enables mussels to support large biomass per unit area, and also creates habitat for other species in these communities^3,4^. The capacity of the byssus to attach to a wide variety of surfaces also enables mussels to expand their habitats by utilizing less competitive surfaces, including artificial underwater constructs such as piers, quay walls, and aquaculture nets^5^. Thus, the byssus is essential to the unique lifestyle of mussels and their roles in marine and freshwater ecosystems.

Components of the byssus have been studied for more than 40 years, and major components, including several collagens, and structural and cuticle proteins, designated as foot proteins (fps), have been discovered mainly in *Mytilus* spp.^2,6^. Gradient distribution of different types of collagens with silk-like (Col-D) and elastic (Col-P) domains as well as a connecting collagen (Col-NG) have been discovered^7^. Six major types of fps (fp-1 to -6) have been discovered and each contains post-translationally modified amino acid residues such as Dopa (3,4-dihydroxyphenylalanine), hydroxyproline, hydroxyarginine, and phosphoserine^2,6^. Although such amino acid modifications, especially the crosslinking of Dopa residues, are thought to be involved in polymerization and insolubilization of fps, detailed functions have yet to be confirmed^2^. Moreover, expansion of these analyses to other mussel species suggest that byssus formation mechanisms are unexpectedly diverse among species. For example, fp-1 from the green mussel, *Perna viridis* (Pvfp-1), less dependent on Dopa-related crosslinking^8,9^, although the corresponding protein of *Myilus edulis* (Mefp-1) contains many Dopa residues^2^.

In this study, we performed whole genome sequencing of the green mussel, *P. viridis* (Fig. 1) to understand physiological systems comprehensively, including byssus formation. The green mussel is a dominant species in Asian tropical and subtropical coastal areas^10–12^, but is also expanding its distribution as an invasive species, even in North and South America^13–17^. The green mussel is a keystone species of coastal ecosystems, forming mussel beds of large biomass and actively ingesting suspended materials and plankton in the water by filter-feeding. In so doing, it transfers the constituents of ingested organisms and their metabolites to organisms higher in the food chain^16,18^. The green mussel is also a major food resource for humans, and is actively cultured in many countries^10,11^. In addition, the green mussel is relatively tolerant of anthropogenic chemical pollutants, concentrating them in the body^11^. It has been proposed as an effective vehicle to monitor environmental pollution^19–22^. Thus, whole genome sequencing of this species is expected to contribute to understanding of its ecology, physiology, and the ecosystems it inhabits.

In this study, we report the high-quality assembly and annotation of whole genome sequences of the green mussel. We also collected transcriptomic data from six major tissues, including the foot, and selected genes expressed at significantly higher levels in the foot than in five other tissues, based on Z-scores. Using genome sequence information with the transcriptomic data, we identified genes involved in byssus formation. Although transcriptomic and proteomic studies have been reported in this species previously^23,24^, the combination of genomic and foot-specific transcriptomic analyses were expected to yield information specific to characteristics of the byssus. After confirming that our data contained genes encoding known fps and collagens, we listed genes exclusively expressed in the foot, the site of byssus production, to find more components involved in byssus formation. As the list included various proteinase inhibitors, defense proteins, and lectins, in addition to structural proteins, such as fps and collagens and their processing enzymes, we propose that byssus formation includes not only construction of resilient structures, but also genes for protection of the byssus.

## Results and discussion

### Results of the sequencing and assembly

A wild green mussel collected in Enoshima, Kanagawa, Japan was used for genome sequencing. By sequencing one paired-end library and 4 mate-paired libraries using an Illumina HiSeq2500 platform, trimmed sequences were obtained (Supplementary Table S1). Analyses of Illumina data with Genome Scope^25^ estimated the genome size at 726 Mb (Supplementary Fig. S1), which is less than half the size of genomes from other mussel species, *Mytilus galloprovincialis* (1.28 Gb), *Mytilus coruscus* (1.90 Gb), *Bathymodiolus platifrons* (1.66 Gb), *Modiolus philippinarum* (2.63 Gb), and *L. fortunei* (1.67 Gb)^26–29^. Genome Scope also estimated heterozygosity at 0.63%, and repetitive portions of the genome at 19.57% (Supplementary Fig. S1). The low heterozygosity may represent a founder effect, since the sampling site, Enoshima, was first colonized by this species only in 1988^30^, and the population may have developed from a limited number of individuals. Sequences were assembled into 15,933 scaffolds (Table 1), of which the longest was 25,893,196 bp. The N50 size was 4,106,945 bp. These statistics suggest that the contiguity of this genome surpasses those of other mussel species (Table 1). From the assembled genome sequences, 24,293 genes were predicted. This predicted gene number was lower than those of other mussel species (Table 2). We think that this is mainly because many predicted genes are disrupted in these species due to fragmented genomes. In fact, the total exon length was comparable to those of *B. platifrons* and *M. philippinarum* (Table 2) and the predicted gene number (24,293) is comparable to those of scallops and oysters (24,521 and 29,738, respectively)^31,32^*.* The total intron length was shorter than those of the two foregoing species, which may reflect the smaller genome size. Evaluation of genome assembly and gene prediction completeness with Benchmarking Universal Single-Copy Orthologs (BUSCO)^33^ using 978 metazoan genes, scored 99.4%, indicating high completeness of the assembly and annotation (Table 3).

**Table 1:** Comparison of the sequencing and assembly statistics of the green mussel *Perna viridis* in the present study with those in other Mytilid mussels reported previously.

Species	*Perna viridis*	*Mytilus galloprovincialis*	*Mytilus coruscus*	*Bathymodiolus platifrons*	*Modiolus philippinarum*	*Limnoperna fortunei*
Total length (bp)	731.8 M	1282.2 M	1903.8 M	1659.3 M	2629.6 M	1673.1 M
Scaffold number	15,933	10,577	10,484	65,664	74,575	20,548
N50 (bp)	4.10 M	0.21 M	0.90 M	0.34 M	0.10 M	0.31 M
L50	49	1904	509	1261	7755	1481
GAP rate	2.30%	2.32%	0.00%	11.80%	4.80%	0.23%

**Table 2:** Comparison of the gene annotation statistics of the green mussel *Perna viridis* in the present study with those of other Mytilid mussels reported previously.

Species	*Perna viridis*	*Mytilus galloprovincialis*	*Mytilus coruscus*	*Bathymodiolus platifrons*	*Modiolus philippinarum*	*Limnoperna fortunei*
Gene number	24,293	60,302	58,249	33,584	36,549	60,717
Exon number per gene	8.0	5.3	5.0	5.2	4.5	3.7
Single exon genes	2915	15,720	15,147	5900	8686	17,705
Total exon length (bp)	37.5 M	73.9 M	74.6 M	37.4 M	38.7 M	67.7 M
Mean exon length (bp)	192.7	230.3	254.0	212.8	233.4	298.2
Mean CDS length (bp)	1544.2	1226.9	1281.1	1114.8	1060.0	916.8
Total intron length (bp)	247.7 M	430.5 M	549.2 M	291.1 M	358.5 M	583.6 M
Mean intron length (bp)	1454.3	1652.7	2331.6	2045.2	2770.0	3511.5
GT-AG splicing site	99.5%	99.5%	99.7%	99.0%	99.0%	99.5%

**Table 3:** Comparison of statistics of BUSCO analysis of the green mussel *Perna viridis* in the present study with those of other Mytilid mussels reported previously.

Species	*Perna viridis* (%)	*Mytilus galloprovincialis* (%)	*Mytilus coruscus* (%)	*Bathymodiolus platifrons* (%)	*Modiolus philippinarum* (%)	*Limnoperna fortunei* (%)
Complete	99.4	85.5	81.6	84.7	70.4	54.3
Complete (Single)	98.3	76.6	78.0	83.0	67.0	46.8
Complete (Double)	1.1	8.9	3.6	1.7	3.4	7.5
Fragment	0.4	5.9	3.9	10.4	17.3	23.3
Missing	0.2	8.5	14.5	4.9	12.4	22.4

### Domains amplified in the green mussel genome

According to the method reported in a previous study^27^, Pfam domains whose numbers are increased in the green mussel genome were searched. As a result, numbers of six domains, lectin C-type domain, WAP (Whey Acidic Protein)-type 'four-disulfide core', extracellular link domain, dual specificity phosphatase catalytic domain, KR domain, and tyrosine phosphatase family domain were found increased (Supplementary Table S2). The significance of the domains with increased number will be discussed below.

### Phylogenetic analyses using orthogroups

**Figure 2:**
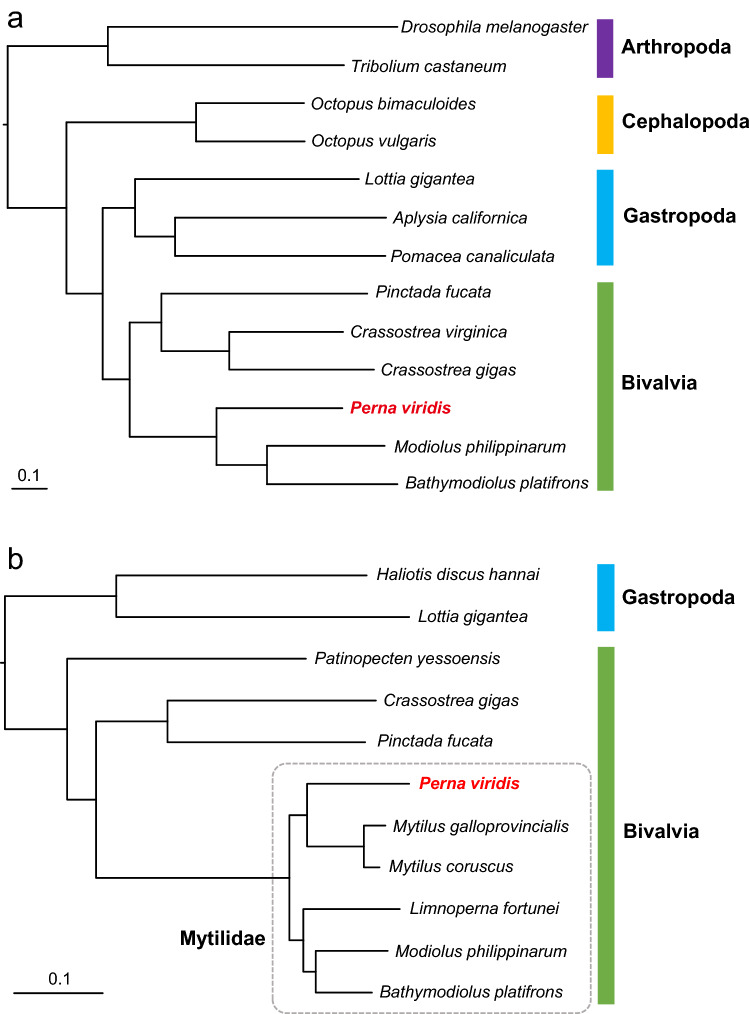
Maximum likelihood (ML) trees of mollusks and bivalves constructed using orthogroups developed from whole-genome sequences. (**a**) phylogenetic tree of mollusks, constructed using 805 genes selected from genome sequences. *Tribolium castaneum* and *Drosophila melanogaster* were used as outgroups. (b) phylogenetic tree of bivalves constructed using 153 genes selected from genome sequences. *Lottia gigantea* and *Haliotis discus hannai* were used as an outgroup. All nodes have 100% bootstrap support after 100 replications.

Annotated genomes are available for 11 mollusk species (Supplementary Table S3), and a relatively high number of genes are predicted from them. Using those 11 genomes, with genomes of two insects (*Tribolium castaneum* and *Drosophila melanogaster*) as outgroups, orthologous group (OG) clustering was performed using OrthoFinder and resulted in 21,139 orthogroups (OGs) from the 13 genomes. Within the 21,139 OGs, 845 single-copy OGs were selected for phylogenomic analysis. Among them, using 805 OGs in which 30 or more amino acids were successfully aligned, phylogenetic analysis was performed (Fig. 2a). As a result, a maximum likelihood (ML) tree with high bootstrap support was constructed. The tree indicates that bivalves are more closely related to gastropods than to cephalopods. Among the hypotheses regarding mollusk phylogeny, our results support the Aculifera-Conchifera hypothesis^34–36^. The phylogeny of Mytilids is also of interest. A phylogenetic tree, including recently published data of *Mytilus galloprovincialis* and *M. coruscus*, was constructed using an alignment of 153 single-copy OGs (Fig. 2b). The result clearly indicated that position *Perna* is phylogenetically closer to *Mytilus* than to other genera, including *Bathymodiolus*, *Modiolus*, and *Limnoperna*. The present result is consistent with phylogeny based on mitochondrial and ribosomal DNA, and transcriptome sequences reported previously^27,37,38^, although the position of *Limnoperna* is inconsistent with a transcriptomic tree reported previously^27^.

### Search for byssal collagen genes

Three major pre-collagen genes reported by Zhang et al.^23^ were discovered among the genome scaffolds. Col-D and Col-NG genes were identified close together on the same scaffold (pvir_s00637g8 and pvir_s00637g10), suggesting generation of the two genes through tandem duplication (Supplementary Figs. S2 and S3). Interestingly, pvir_s00637g9, the gene between the two collagen genes encodes a highly glycine-rich protein, but it is not a collagen because the glycine residues are not arranged in a characteristic G-X-Y repeat. Col-P^23^ corresponded to pvir_s00107g4, although there are some differences in predicted N- and C-terminal sequences (Supplementary Fig. S4).

### Search for genes of known major fps

Genes encoding major fps reported in the green mussel, i.e., those for Pvfp-1 through -6 were searched in the green mussel genome scaffolds using previously reported sequences^23,24^ as queries (Table 4). Known sequences of Pvfp-1 were aligned to different parts of the protein encoded by a single gene, s00107g6, identified in this study (Supplementary Fig. S5). Fp-2 sequences previously identified (AGZ84282) corresponded to the carboxyl terminal part of the protein encoded by pvir_s01028g36 (Supplementary Fig. S6). The whole coding region of Pvfp-2 predicted in this study encoded 23 EGF-like repeats (Supplementary Fig. S7a). A fp-3 sequence, AGZ84284, previously reported, was completely matched to part of the protein encoded by pvir_s136476g25 (Supplementary Fig. S8). Other known fp-3 sequences, AGZ84285, and GGRR01022672, also corresponded to the protein encoded by pvir_s136476g26. The neighboring gene, pvir_s136476g24 also encodes a similar protein (Supplementary Fig. S8). Thus, Pvfp-3 is likely to exist as a multiple copy gene, as suggested in *M. galloprovincialis* fp-3^39^, and the copies were generated through tandem duplications. Pvfp-4, reported by Zhang et al.^23^ (GGRR01022802) almost matched the protein encoded by pvir_s00068g44 (Supplementary Fig. S9). Three genes, pvir_s001219b108, pvir_s001219g109, and s001219g110, arranged in tandem on the same scaffold, encode previously reported Pvfp-5 sequences (Table 4 and Supplementary Fig. S10). Thus, Pvfp-5 is a multicopy gene family generated by tandem duplications. Pvfp-6 reported in previous studies, GGRR01025060 and AGZ84283, matched pvir_s00010g6, except for two amino acid substitutions (Supplementary Fig. S11).

**Table 4:** Foot protein genes identified in the whole genome sequence of *Perna viridis* using previously known foot protein sequences as query.

Gene name	Putative *Perna* gene	Length (aa)	GenBank ID of *Perna* sequence	Length (aa)
fp-1	s00107g6	321	GGRR01024513	71
			AGZ84280	131
			AGZ84281	65
fp-2	s01028g36	907	AGZ84282	322
fp-3	s136476g25	86	GGRR01022782	70
			AGZ84284	70
	s136476g26	70	AGZ84285	70
fp-4	s00068g44	349	GGRR01022802	565
fp-5	s01219g108	113	AGZ84277, AGZ84275, AGZ84276^a^	99, 88, 139
	s01219g109	140	GGRR01024248, AGZ84278, AGZ84276^a^	140, 140, 139
	s01219g110	176	AGZ84279	176
fp-6	s00010g6	122	GGRR01025060	122
			AGZ84283	122

Peptide alignments are shown in Supplementary Figs S5 to S11.

^a^Partial matching to the two genes.

Dominant genes exclusively expressed in the foot.

**Figure 3:**
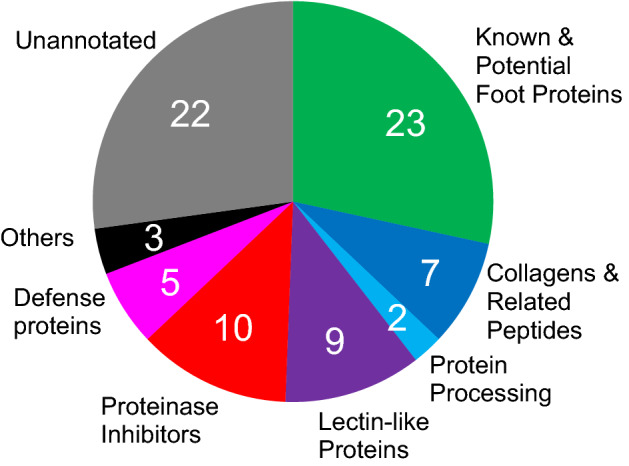
Classification of 81 Highly Expressed genes in the Foot (HEFs) in *Perna viridis*. Numbers are gene numbers in each category.

The bivalve foot is a multifunctional organ that functions as a sensor and a locomotor organ. However, for mussels, byssus synthesis is its primary role, and much of the foot is occupied by glands that secrete byssal components and the ventral groove, which is a template for the byssus^40^. In addition, in *Mytilus*, genes for some byssal components are expressed exclusively in the foot^41–43^. Therefore, we compiled transcripts exclusively detected in the foot (Z-score > 1.96), which is the point of the present study, compared with previous foot-transcriptome studies in *P. viridis* and *M. californianus*^23,24,44^ and foot transcriptomic and proteomic studies in *M. coruscus*^45^. A list of foot-specifically expressed genes was arranged in descending order of Transcripts Per Million (TPM) (Supplementary Table S4). The top 81 genes were dominated by possible byssus-related genes, so we denominated them Highly Expressed genes in the Foot (HEFs). Most peptides encoded by HEFs are predicted to have signal peptides (Supplementary Table S4). Annotations of listed genes were searched against the Swiss-Prot database (Supplementary Table S4), and 39 HEFs were successfully annotated. HEFs with no hits were subjected to global BLAST and motif searches, and possible functions were predicted for an additional 20 genes. The remaining 22 genes could not be annotated. We categorized the 59 HEFs with annotations or probable functions into 6 groups: known and potential fps (23 genes), collagens and related proteins (7 genes), protein-processing proteins (2 gene), lectin-like proteins (9 genes), proteinase inhibitors (10 genes), biological defense-related proteins (5 genes), and others (3 genes) (Fig. 3). Details of each group are described below.

*Known and potential fps:* All known fp-genes described above (Table 4), except fp-4 with low TPM, were found among 90 HEFs. Many are annotated as Notch or (Proto)Cadherin or no hit by Swiss-Prot searches, because of the lack of mussel-specific genes in the database. Therefore, 10 other genes that are annotated as Notch- or Cadherin-like are categorized as potentially novel fp genes. Among them, two genes, pvir_s01028g37 and pvir_s00819g17 encode proteins consisting mainly of long EGF-like repeats with terminal peptides containing tyrosine residues (Supplementary Fig. S7b,c). Such characteristics are common in fp-2 of *Mytilus*^41^. Especially, the former is tandemly localized with pvir_s01028g36 encoding *P. viridis* fp-2 (Supplementary Fig. S7a); thus, it is likely to be an additional copy of fp-2 generated through tandem duplication, although the number of EGF-like repeats is different. As the other gene, pvir_s00819g17, encodes highly conserved repeats, it should be considered a different class from fp-2. Another 9 genes have structural characteristics similar to fp-5 s, i.e., they contain 1–7 EGF-like repeats (Supplementary Fig. S12). Phylogenetic analysis of these proteins with known fp-5 s (Supplementary Fig. S13) indicated that pvir_s01219g106 and pvir_s01219g107 are additional tandem copies of fp-5 genes. Other Pvfp-5-like proteins, encoded by scaffolds other than pvir_s01219, formed two separate clades: one includes two genes on pir_s00079 and the other comprises those on pvir_s00296, pvir_s02522, and pvir_s136446. Proteins encoded by these genes may be functionally independent from known Pvfp-5 s.

*Collagens and related proteins:* 7 genes were annotated as collagens using Swiss-Prot searches. Among the three major known collagens, Col-D and Col-NG exhibited the highest TPM values. However, pvir_s00107g4, encoding a known Col-P had low TPM. Its neighboring gene, pvir_s00107g5, also contained relatively short G-X-Y repeats and was identified as a proximal thread matrix protein (PTMP)^23^. Three tandem genes, pvir_s00096.g4, pvir_s00096.g5, and pvir_s00096.g6 were novel collagen genes, and they may be important for byssus formation, considering their high expression levels. One remaining gene was actually not a typical collagen because it encoded a protein without G-X-Y repeats. It was annotated as a collagen because it is similar to the non-G-X-Y part of a mammalian collagen; thus, it may interact with collagens.

*Protein processing proteins:* One gene was annotated as a peptidyl-prolyl cis-trans isomerase, which constructs specific helical structures of collagens^46,47^. Protein transport protein (Sec61)-like genes were also included among HEFs.

*Lectin-like proteins:* 9 genes were annotated as C-type lectins or lectin domain-containing proteins. They may participate in biological defense mechanisms^48^, and they may also be involved in specific conformation of fps or collagens by regulating glycosylation, in cooperation with enzymes involved in sugar chain regulation. The sets of genes, pvir_s00346g40/pvir_s00346g41 and pvir_s00466g48/pvir_s00466g49, of which the latter accompanies one similar gene pvir_s00466g47 with lower TPM, were found in tandem, suggesting amplification by tandem duplication. Such amplification of lectin-like genes may be related to the amplification of C-type lectin domains (Supplementary Table S2).

*Proteinase inhibitors:* It is surprising that 10 proteinase inhibitors, including serine protease inhibitors and metalloprotease inhibitors, are included among HEFs, although transcripts for a serine protease inhibitor have been reported previously^24^. If these proteinase inhibitors are deposited in the byssus, they may protect it from attack by proteinases produced by bacteria and other organisms. The resistance of the byssus to various proteinases has been attributed to crosslinking and polymerization^40^. However, inclusion of proteinase inhibitors may be an additional reason. It is also possible that they protect byssal proteins until crosslinking and polymerization are complete, although it is still possible that these proteinase inhibitors may be protecting the foot tissue. Two HEFs, pvir_s00544g11 and pvir_s00884g60 were found to have WAP-type, four-disulfide core domains, which are amplified in the genome (Supplementary Table S2), but without significant annotation against SwissProt. These genes may encode novel important proteins.

*Biological defense-related proteins:* Five HEFs likely function in self-defense. The protein encoded by pvir_s00401g98 is similar to a *Mytilus* antimicrobial protein. Genes encoding lysozyme and lipopolysaccharide-binding protein are also supposed to be involved in anti-bacterial defense. A CD109-like molecule may also function in self-defense. pvir_s01169g96 encodes a protein containing CUB and sushi domains, which are involved in the complement cascade. Considering their specific expression in the foot, these molecules may be specialized for protection of the byssus.

*Others:* High levels of expression of ornithine decarboxylase antizyme, tubulin, and protein transport protein genes are of interest, but the degree of specificity of expression in the foot is not high. Expression of proteins similar to thrombospondin type-1 domain-containing protein in the foot is interesting, but it is difficult to speculate on their functions in the foot and further study is needed. In addition, 21 HEFs that could not be annotated are expected to be important for byssus formation and maintenance.

Genes expressed exclusively in the foot, but at lower levels.

Other genes in the same 6 categories, but less highly expressed than HEFs, were also identified (Supplementary Table S4). Among them, proteinase inhibitor-like genes were strongly detected, suggesting involvement of many genes to protect the byssus from proteolysis. Some such genes, for example, 11 genes on scaffold pvir_s00124, 4 genes on pvir_s00028, and 2 genes each on pvir_s00161 and pvir_s00234, were found clustered in tandem on the same scaffold, suggesting expansion via tandem duplications, perhaps to achieve bulk production. Eight copies of CD109 antigen-like genes, one of which is included among HEFs, were arranged in tandem on scaffold pvir_s00037.

Many potential enzyme genes are also present. Notably, 11 tyrosinase-like genes were identified. Four of them are on scaffold pvir00137, two are on pvir00219, and two are on pvir_00667, suggesting that tyrosinase genes have been tandemly duplicated. Tyrosinases are involved in Dopa formation and crosslinking processes; thus, they play central roles in byssus formation^2^, and their expansion is quite reasonable. Existence of multiple tyrosinase genes was also detected in the foot of *M. coruscus* in a proteomic analysis^45^.

In addition, a number of genes that are likely involved in byssal protein modification were identified. For example, prolyl 4-hydroxylase-like, protein disulfide-isomerase-like genes, and peptidyl-prolyl cis-trans isomerase-like genes, which promote collagen helix formation^46,47^, were included. Genes annotated as other enzymes, such as serine/threonine protein kinase, phenylalanine-4-hydroxylase, protein phosphatase, bifunctional arginine demethylase and lysyl-hydroxylase, and protein arginine N-methyltransferase are also potentially involved in metabolism and processing of byssal proteins. Moreover, numerous genes were annotated as sugar chain-modification enzymes, many of which act upon mannosyl residues. These genes are also likely involved in byssus formation or function, as the importance of glycosylation has been reported in green mussel fp-1^9,49^, quagga mussel, *Dreissena bugensis* fp-1^50^, and PTMP of *M. galloprovincialis*^51^. In addition, the importance of sugar-mediated binding has been reported in the byssus of the fan shell, *Atrina pectinate*^52^. Some potential sugar modification enzyme genes are also found duplicated in tandem. For example, pvir_s00101g48 and pvir_s00101g50, pvir_s00101g97 and pvir_s00101g99, and pvir_s00130g47 and pvir_s00130g48 are annotated as beta-1,3-galactosyltransferase, glycoprotein_3-alpha-L-fucosyltransferase, and glycosyltransferase-25-like genes, respectively (Supplementary Table S4). Such genes may have been amplified for bulk production. Notably, some peptide motifs in the green mussel genome (Supplementary Table S2) are probably related to protein processing.

Two genes annotated as superoxide dismutases (SOD) (pvir_s00007g27 and pvir_s00007g29), which are tandemly located in the genome, were also expressed exclusively in the foot (Supplementary Table S4). While 12 SOD-like genes were detected in the green mussel genome (Supplementary Table S5), 10 other genes were not foot-specific. Antioxidant activity is reportedly important for byssus formation, and fp-6 has antioxidant activity^53^. These two foot-specific SOD genes may contribute to formation of the byssus as antioxidants.

## Conclusion

**Figure 4:**
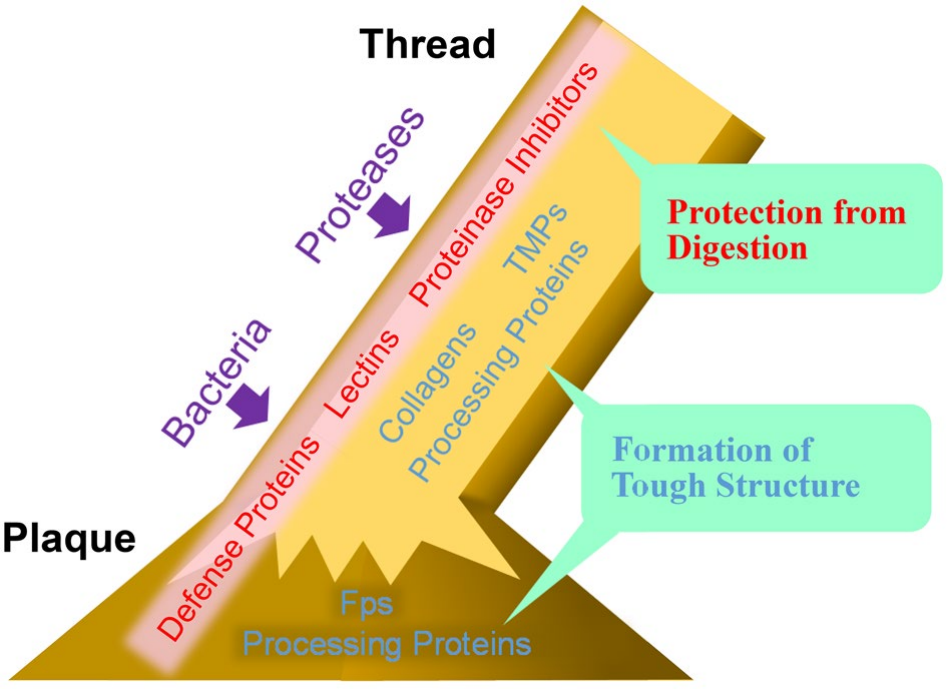
Strategy of the green mussel to form a tough, durable byssus, suggested by whole-genome and transcriptome analyses. Major genes expressed exclusively in the foot were classified into two major categories: genes encoding structural proteins (collagens, TMPs, and foot proteins) and processing proteins, including modification and polymerization enzymes and transport proteins, and those encoding proteins that protect the byssus from attack by bacteria and proteinases.

In conclusion, the results of foot-specific RNA-seq analysis suggest that two mechanisms are involved in creating a tough, durable byssus (Fig. 4). One is to employ resilient materials, including structural proteins such as collagens, fps, and TMPs, and modifying components, such as enzymes and lectins, to construct specific structures. The other mechanism is to protect byssal proteins against proteases and bacteria, in which defense proteins including lysozyme, proteinase inhibitors, and possibly lectins are involved. Previous studies on byssus formation mechanisms have focused mainly on the former. However, the number of genes involved in defense mechanisms identified in this study indicate that mussels devote considerable energy to the latter, which is likely the key to byssus durability. In addition, we propose that identification of genes expressed exclusively in the foot is a useful approach to study the complicated process of byssus formation and maintenance.

## Methods

### Mussels

Specimens of *Perna viridis* were collected at Enoshima, Fujisawa, Japan, on August 27, 2018, and maintained in a plastic tank at the Atmosphere and Ocean Research Institute, The University of Tokyo, using natural seawater, until use. Foot, gill, mantle, adductor muscle, and testes were dissected from a male specimen with a shell length of approximately 4 cm. Dissected tissues were kept at -80°C until DNA or RNA extraction.

### Genomic DNA extraction, sequencing, and de novo assembly

Genomic DNA was extracted from the testis using a Mag attract HMW DNA kit (Qiagen, Santa Clarita, CA, USA). Paired-end (250 bp) and mate-pair libraries (3, 6, 10, and 15 kb) were constructed using a TruSeq DNA PCR-Free LT Sample Prep Kit and a Nextera Mate Pair Sample Prep Kit (Illumina, San Diego, CA, USA). Sequencing employed an Illumina HiSeq2500. We generated 731,873,830 bp of raw sequence data for *de novo* genome assembly. Genome assembly into scaffolds was performed using the Platanus v1.2.4 assembler^54^ after removal of adapter sequences (Platanus_trim and Platanus_internal_trim). Contig assembly was performed using only the PE library, and then scaffolding and gap closure were performed using all libraries. Genome Scope^25^ was used for genome size and heterozygosity analyses. Generated scaffolds were named in order of length as pvir_s00001, starting with the longest.

### Gene annotation

Genes on genome scaffolds were predicted by combining RNA-seq-based prediction results, homology-based prediction results for related species, and *ab initio* prediction results. RNA-seq based prediction utilized both the assembly-first method and the mapping-first method. For the assembly-first method, RNA-seq data were assembled using Trinity^55^ and Oases^56^. Then, assembled contigs were splice-mapped with GMAP^57^. For the mapping-first method, RNA-seq data were mapped to genome scaffolds using HISAT2^58^. Then gene sets were predicted with StringTie^59^ from mapped results. Details of RNA-seq data are described below. In terms of homology-based prediction, amino acid sequences of *Patinopecten yessoensis*, *Lottia gigantea*, *Crassostrea giga*, *Modiolus philippinarum*, *Bathymodiolus platifrons*, and *Mytilus galloprovincialis*^26,31,32,60,61^, were spliced-mapped to genome scaffolds using Spaln^62^, and gene sets were predicted. For *ab initio* prediction, first, training sets were selected from RNA-seq based prediction results. Then AUGUSTUS^63^ was trained using this set. SNAP^64^ was also used. Finally, all predicted genes were combined using an in-house merging tool. Predicted genes were functionally annotated with NCBI Swiss-Prot (October, 2019) using BLAST^65^ with an e-value cutoff of 1e^−5^. Each gene was named ‘g’ plus a number from one end of each scaffold, e.g., pvir_s00001.g1 is the first gene of scaffold pvir_s00001. Transcriptomic and proteomic completeness for each longest transcript were evaluated using BUSCO v3.0.2^33^ with the metazoan dataset. Search for Pfam domains whose numbers are expanded in the green mussel genome were conducted as described previsouly^27^.

### RNA extraction, sequencing, and expression level detection

Total RNA was extracted from seven tissues (adductor muscle, foot, gill, testis, intestine, kidney, and mantle) using TRIzol reagent, and each library was prepared using a TruSeq Standed mRNA prep kit (Illumina) and sequenced on a Nova Seq 6000 platform (Illumina) with 100-bp paired-ends by Macrogen Corporation, Japan. Low-quality reads (Q<20 and length <25 bp) were trimmed with CUTADAPT v1.18^66^. Then, cleaned reads were mapped to *Perna* gene models with SALMON v0.14.1^67^ using default settings. Expression levels were expressed as transcripts per million (TPM).

### Phylogenetic analysis

For comparison of orthologs, we used genomes of several metazoan species (Supplementary Table S3). Gene models of mussels (*B. platifrons* and *M. philippinarum*) were downloaded from DRYAD (https://datadryad.org)^27^. The genome of the pearl oyster (*Pinctada fucata*) was downloaded from the Genome browser of the Marine Genomics Unit at OIST (Okinawa Institute of Science and Technology) (https://marinegenomics.oist.jp/). Genomes of other taxa were downloaded from NCBI Reference Sequence (RefSeq). Gene models for genomes downloaded from GenBank assembly and RefSeq were generated with Gffread^68^ and were translated to amino acids using TransDecoder v5.5.0^69^. Then, the longest transcript variant for each gene model was retained. Ortholog groups (OGs) with no gaps were identified from translated longest sequences using OrthoFinder v2.3.3^70^ with default settings. Identified OGs were annotated with the human proteome, downloaded from Uni-Prot (October, 2019) or NCBI Swiss-Prot (October, 2019) using BLAST^65^ with e-value cutoff of 1e^−5^. All sequences were aligned using MAFFT v7.429^71,72^, and gaps in alignments were removed using trimAL v1.2^73^. Then aligned amino acid sequences of each OG were concatenated. Maximum likelihood (ML) analyses were performed using RAxML v8.2.11^74^ with the PROTGAMMAAUTO option.

For comparison of orthologs from bivalves, we used protein sequences of 9 bivalves (*P. viridis*, *P. yessoensis*, *C. gigas*, *P. fucata*, *L. fortunei*, *M. philippinarum*, *B. platifrons*, *M. galloprovincialis* and *M. coruscus*) and 2 snails (*Haliotis discus hannai*, *L. gigantea*). Protein sequences of *L. fortunei* and *H. discus hannai* were downloaded from GigaDB (http://gigadb.org/). Identification of orthologous groups of proteins was performed as follows. First, protein sequences from all species were grouped into gene families and 153 ortholog groups were extracted with a one-to-one relationship across all species, using Proteinortho ^75^. For each group, multiple alignments were performed with MAFFT^71^ and sites containing gaps (“−”) or ambiguous characters (“X”) were excluded. All alignments were concatenated, and 34,223 amino acid sites were used for phylogenetic analysis. A phylogenetic tree was constructed with RAxML (version 8.2.12)^74^. Here, we applied the JTT substitution matrix with a gamma model of rate heterogeneity (-m PROTGAMMAJTT). The ML tree of fp-5-like sequences was also constructed using MAFFT^71^ and RAxML v8.2.11^74^ with the PROTGAMMAAUTO option.

### Exploring putative byssal proteins

Putative foot protein genes in the genome assembly were searched with BLAST using previously reported *Perna* foot proteins^23,24^ and mussel foot proteins downloaded from NCBI.

### Searching for genes exclusively expressed in the foot

TPMs of all genes were standardized using Z-scores across TPM over all organs. Predicted genes for which TPM counts in the foot were significantly higher than those of the other six organs were listed (Z-score > 1.96; Supplementary Table S4). From the listed genes, those with TPM counts higher than 550 were selected as HEFs. HEF genes with no hit against SwissProt were subjected to TBLASTN searches in the DNA Data Bank of Japan and Prosite (https://prosite.expasy.org/), using default settings. Signal peptides and motifs in fp-like proteins were identified using SignalP-5.0 (http://www.cbs.dtu.dk/services/SignalP/) and Prosite, respectively. SOD genes were picked up according to BLAST annotation against SwissProt.

## Supplementary information

Supplementary Figures.

Supplementary Table S1.

Supplementary Information Table S2.

Supplementary Information Table S3.

Supplementary Information Table S4.

Supplementary Information Table S5.

## Data Availability

The *Perna viridis* genome sequence can be accessed at DDBJ and Bioproject (NCBI) as PRJDB10567, which links to the Sequence Read Archive for all genome raw and assembled scaffold (nucleotide) data under BioSamples SAMD00247165.
