# Supplementary material for: Genomics and Transcriptomics of the green mussel explain the durability of its byssus
Source: Sci Rep. 2021 Mar 16;11:5992. doi: 10.1038/s41598-021-84948-6 (PMC7971044; doi:10.1038/s41598-021-84948-6)
Supplement: Supplementary file 1 — Supplementary Figures. [file 41598_2021_84948_MOESM1_ESM.pptx]

## Slide 1
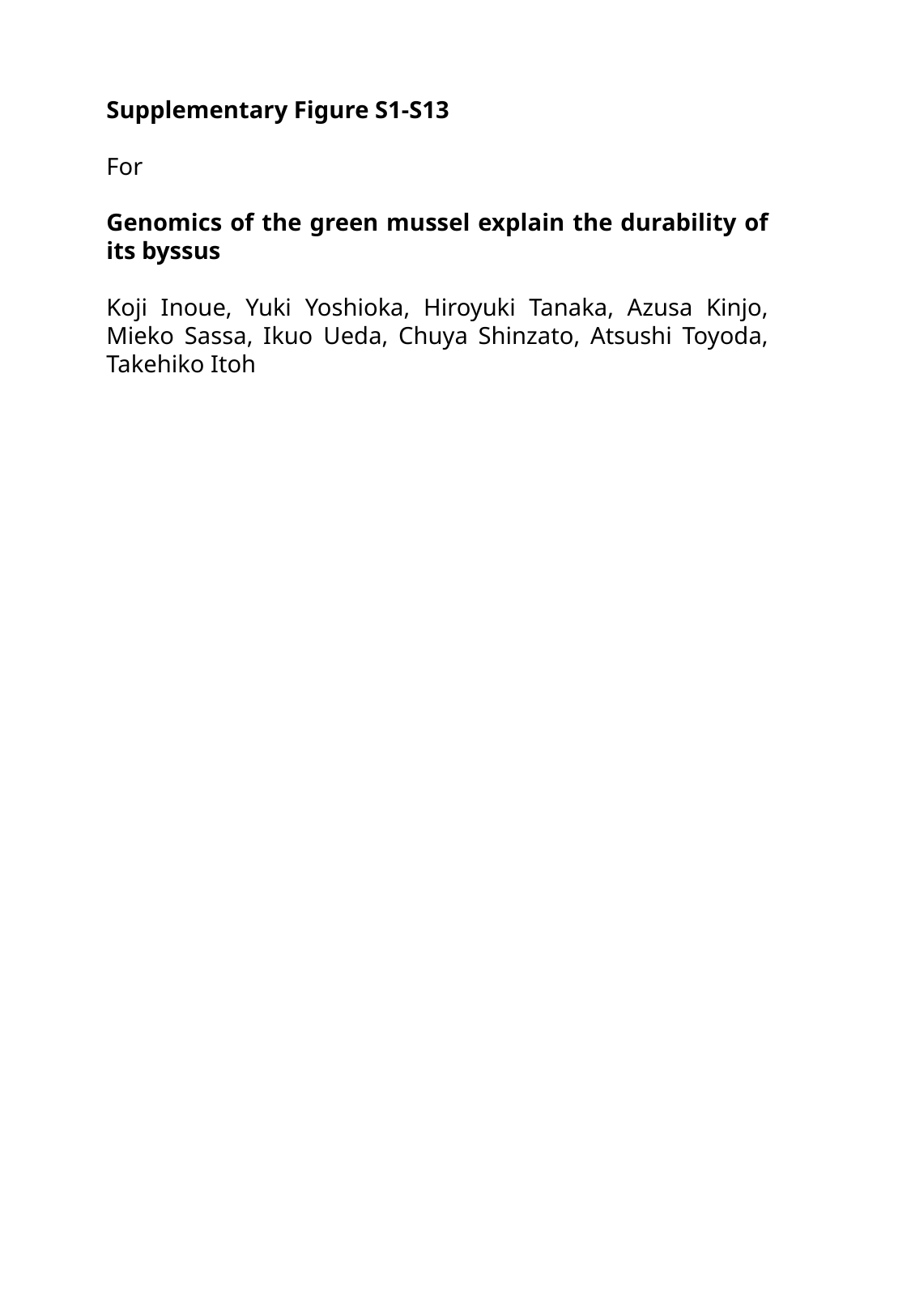

Supplementary Figure S1-S13
For
Genomics of the green mussel explain the durability of its byssus
Koji Inoue, Yuki Yoshioka, Hiroyuki Tanaka, Azusa Kinjo, Mieko Sassa, Ikuo Ueda, Chuya Shinzato, Atsushi Toyoda, Takehiko Itoh

## Slide 2
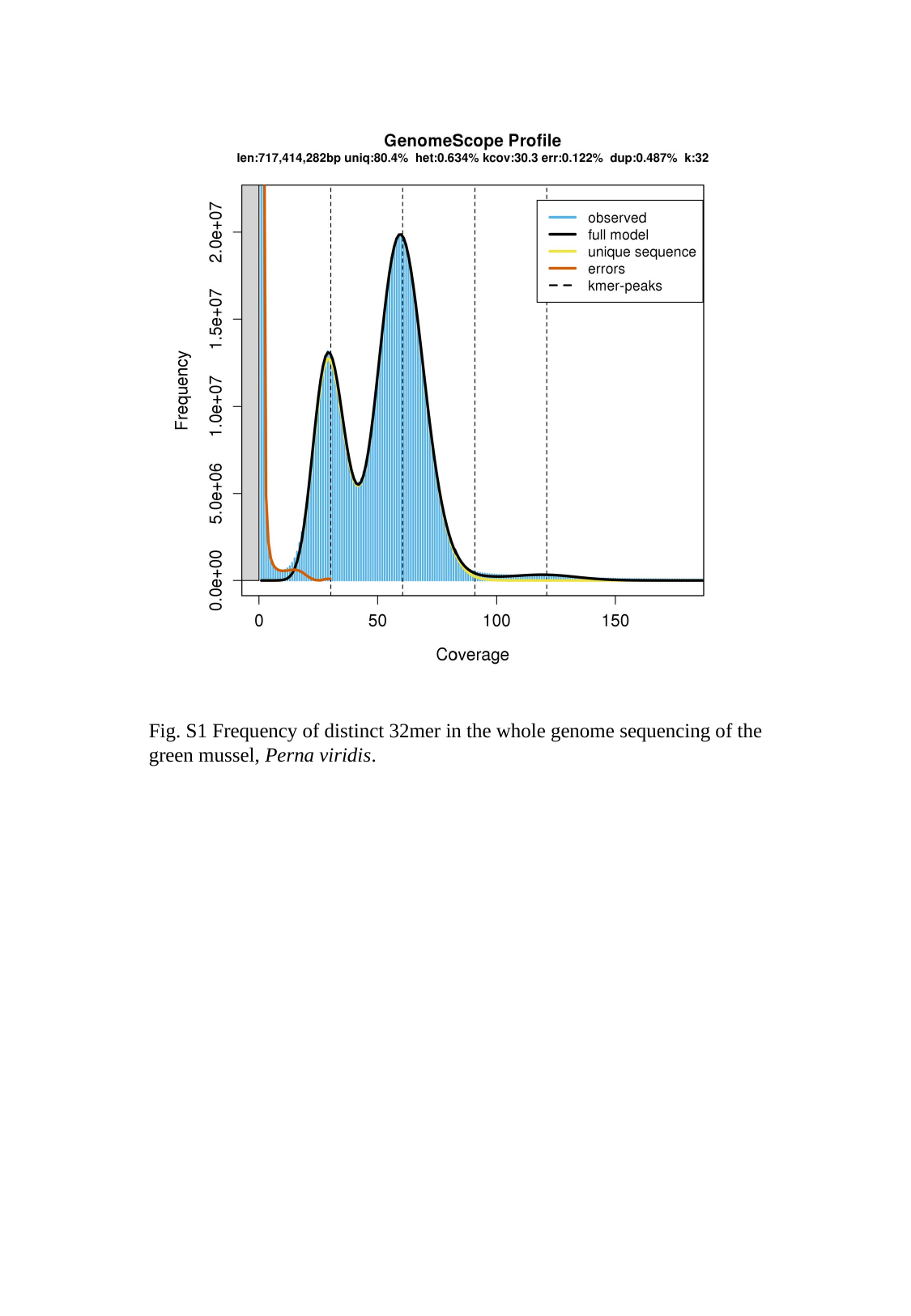

Fig. S1 Frequency of distinct 32mer in the whole genome sequencing of the green mussel, Perna viridis.

## Slide 3
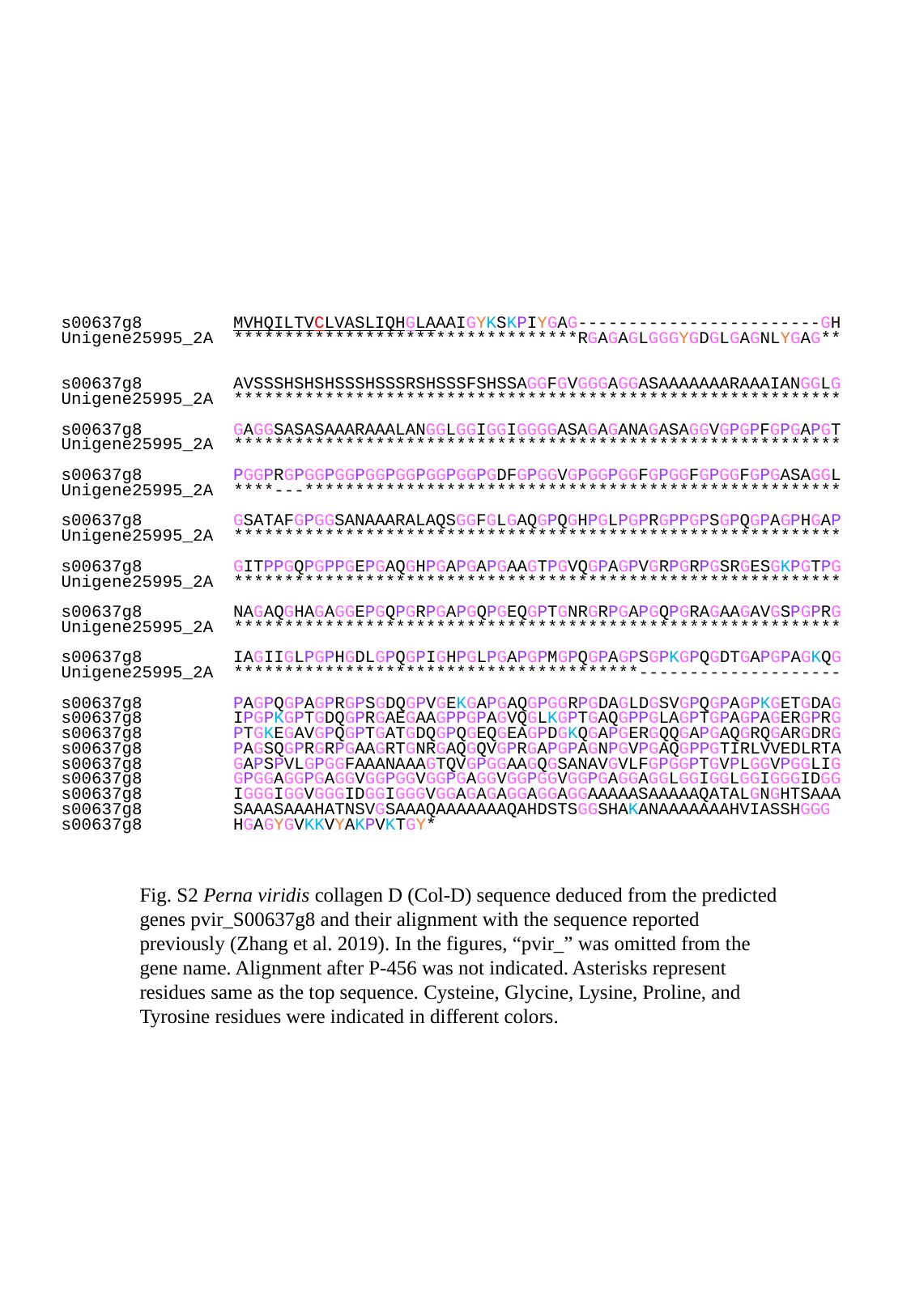

s00637g8 	 MVHQILTVCLVASLIQHGLAAAIGYKSKPIYGAG------------------------GH
Unigene25995_2A **********************************RGAGAGLGGGYGDGLGAGNLYGAG**
s00637g8 	 AVSSSHSHSHSSSHSSSRSHSSSFSHSSAGGFGVGGGAGGASAAAAAAARAAAIANGGLG
Unigene25995_2A ************************************************************
s00637g8 	 GAGGSASASAAARAAALANGGLGGIGGIGGGGASAGAGANAGASAGGVGPGPFGPGAPGT
Unigene25995_2A ************************************************************
s00637g8 	 PGGPRGPGGPGGPGGPGGPGGPGGPGDFGPGGVGPGGPGGFGPGGFGPGGFGPGASAGGL
Unigene25995_2A ****---*****************************************************
s00637g8 	 GSATAFGPGGSANAAARALAQSGGFGLGAQGPQGHPGLPGPRGPPGPSGPQGPAGPHGAP
Unigene25995_2A ************************************************************
s00637g8 	 GITPPGQPGPPGEPGAQGHPGAPGAPGAAGTPGVQGPAGPVGRPGRPGSRGESGKPGTPG
Unigene25995_2A ************************************************************
s00637g8 	 NAGAQGHAGAGGEPGQPGRPGAPGQPGEQGPTGNRGRPGAPGQPGRAGAAGAVGSPGPRG
Unigene25995_2A ************************************************************
s00637g8 	 IAGIIGLPGPHGDLGPQGPIGHPGLPGAPGPMGPQGPAGPSGPKGPQGDTGAPGPAGKQG
Unigene25995_2A ****************************************--------------------
s00637g8 	 PAGPQGPAGPRGPSGDQGPVGEKGAPGAQGPGGRPGDAGLDGSVGPQGPAGPKGETGDAG
s00637g8 	 IPGPKGPTGDQGPRGAEGAAGPPGPAGVQGLKGPTGAQGPPGLAGPTGPAGPAGERGPRG
s00637g8 	 PTGKEGAVGPQGPTGATGDQGPQGEQGEAGPDGKQGAPGERGQQGAPGAQGRQGARGDRG
s00637g8 	 PAGSQGPRGRPGAAGRTGNRGAQGQVGPRGAPGPAGNPGVPGAQGPPGTIRLVVEDLRTA
s00637g8 	 GAPSPVLGPGGFAAANAAAGTQVGPGGAAGQGSANAVGVLFGPGGPTGVPLGGVPGGLIG
s00637g8 	 GPGGAGGPGAGGVGGPGGVGGPGAGGVGGPGGVGGPGAGGAGGLGGIGGLGGIGGGIDGG
s00637g8 	 IGGGIGGVGGGIDGGIGGGVGGAGAGAGGAGGAGGAAAAASAAAAAQATALGNGHTSAAA
s00637g8 	 SAAASAAAHATNSVGSAAAQAAAAAAAQAHDSTSGGSHAKANAAAAAAAHVIASSHGGG
s00637g8 	 HGAGYGVKKVYAKPVKTGY*
Fig. S2 Perna viridis collagen D (Col-D) sequence deduced from the predicted genes pvir_S00637g8 and their alignment with the sequence reported previously (Zhang et al. 2019). In the figures, “pvir_” was omitted from the gene name. Alignment after P-456 was not indicated. Asterisks represent residues same as the top sequence. Cysteine, Glycine, Lysine, Proline, and Tyrosine residues were indicated in different colors.

## Slide 4
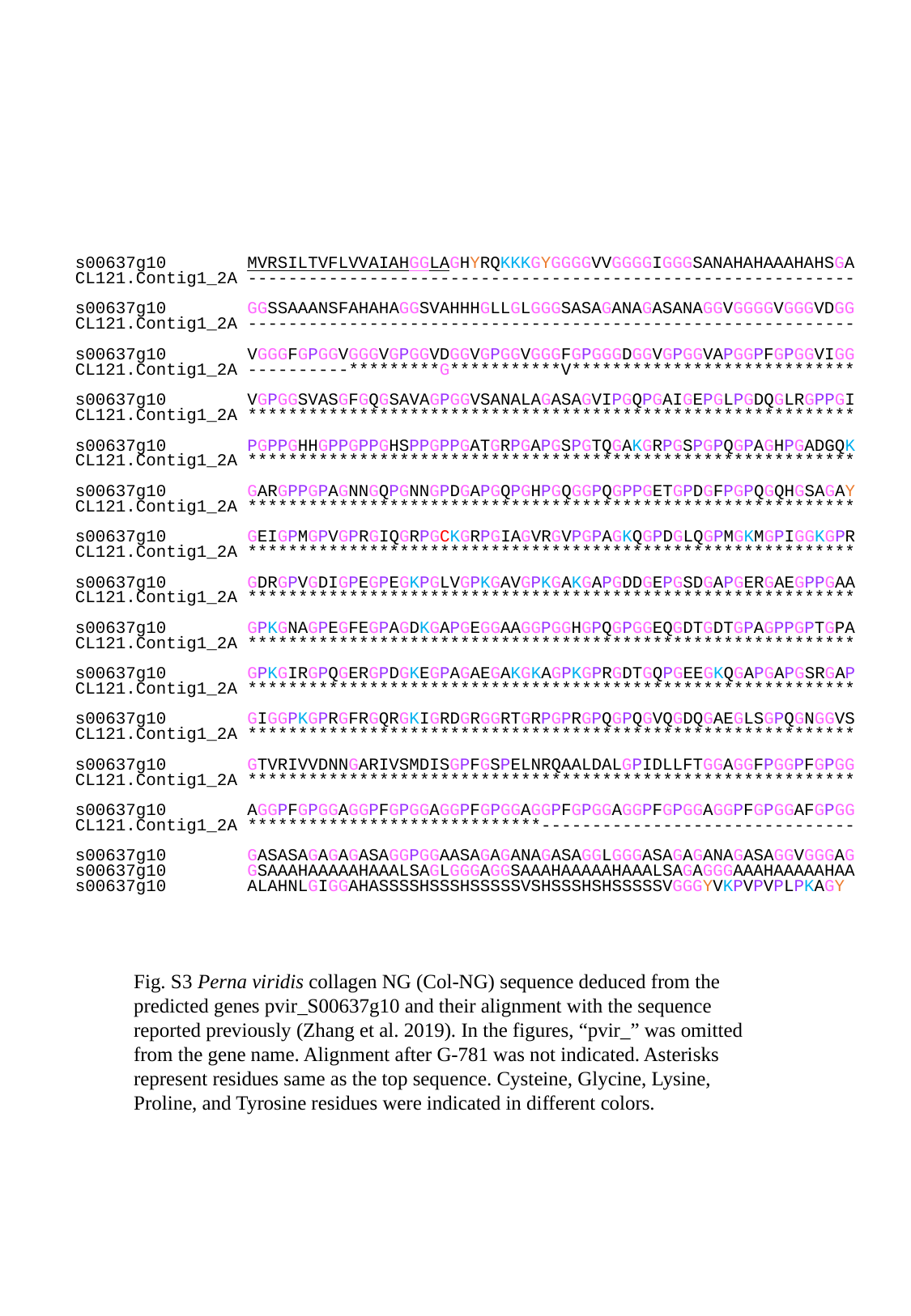

s00637g10 	 MVRSILTVFLVVAIAHGGLAGHYRQKKKGYGGGGVVGGGGIGGGSANAHAHAAAHAHSGA
CL121.Contig1_2A ------------------------------------------------------------
s00637g10 	 GGSSAAANSFAHAHAGGSVAHHHGLLGLGGGSASAGANAGASANAGGVGGGGVGGGVDGG
CL121.Contig1_2A ------------------------------------------------------------
s00637g10 	 VGGGFGPGGVGGGVGPGGVDGGVGPGGVGGGFGPGGGDGGVGPGGVAPGGPFGPGGVIGG
CL121.Contig1_2A ----------*********G***********V****************************
s00637g10 	 VGPGGSVASGFGQGSAVAGPGGVSANALAGASAGVIPGQPGAIGEPGLPGDQGLRGPPGI
CL121.Contig1_2A ************************************************************
s00637g10 	 PGPPGHHGPPGPPGHSPPGPPGATGRPGAPGSPGTQGAKGRPGSPGPQGPAGHPGADGQK
CL121.Contig1_2A ************************************************************
s00637g10 	 GARGPPGPAGNNGQPGNNGPDGAPGQPGHPGQGGPQGPPGETGPDGFPGPQGQHGSAGAY
CL121.Contig1_2A ************************************************************
s00637g10 	 GEIGPMGPVGPRGIQGRPGCKGRPGIAGVRGVPGPAGKQGPDGLQGPMGKMGPIGGKGPR
CL121.Contig1_2A ************************************************************
s00637g10 	 GDRGPVGDIGPEGPEGKPGLVGPKGAVGPKGAKGAPGDDGEPGSDGAPGERGAEGPPGAA
CL121.Contig1_2A ************************************************************
s00637g10 	 GPKGNAGPEGFEGPAGDKGAPGEGGAAGGPGGHGPQGPGGEQGDTGDTGPAGPPGPTGPA
CL121.Contig1_2A ************************************************************
s00637g10 	 GPKGIRGPQGERGPDGKEGPAGAEGAKGKAGPKGPRGDTGQPGEEGKQGAPGAPGSRGAP
CL121.Contig1_2A ************************************************************
s00637g10 	 GIGGPKGPRGFRGQRGKIGRDGRGGRTGRPGPRGPQGPQGVQGDQGAEGLSGPQGNGGVS
CL121.Contig1_2A ************************************************************
s00637g10 	 GTVRIVVDNNGARIVSMDISGPFGSPELNRQAALDALGPIDLLFTGGAGGFPGGPFGPGG
CL121.Contig1_2A ************************************************************
s00637g10 	 AGGPFGPGGAGGPFGPGGAGGPFGPGGAGGPFGPGGAGGPFGPGGAGGPFGPGGAFGPGG
CL121.Contig1_2A *****************************-------------------------------
s00637g10 	 GASASAGAGAGASAGGPGGAASAGAGANAGASAGGLGGGASAGAGANAGASAGGVGGGAG
s00637g10 	 GSAAAHAAAAAHAAALSAGLGGGAGGSAAAHAAAAAHAAALSAGAGGGAAAHAAAAAHAA
s00637g10 	 ALAHNLGIGGAHASSSSHSSSHSSSSSVSHSSSHSHSSSSSVGGGYVKPVPVPLPKAGY
Fig. S3 Perna viridis collagen NG (Col-NG) sequence deduced from the predicted genes pvir_S00637g10 and their alignment with the sequence reported previously (Zhang et al. 2019). In the figures, “pvir_” was omitted from the gene name. Alignment after G-781 was not indicated. Asterisks represent residues same as the top sequence. Cysteine, Glycine, Lysine, Proline, and Tyrosine residues were indicated in different colors.

## Slide 5
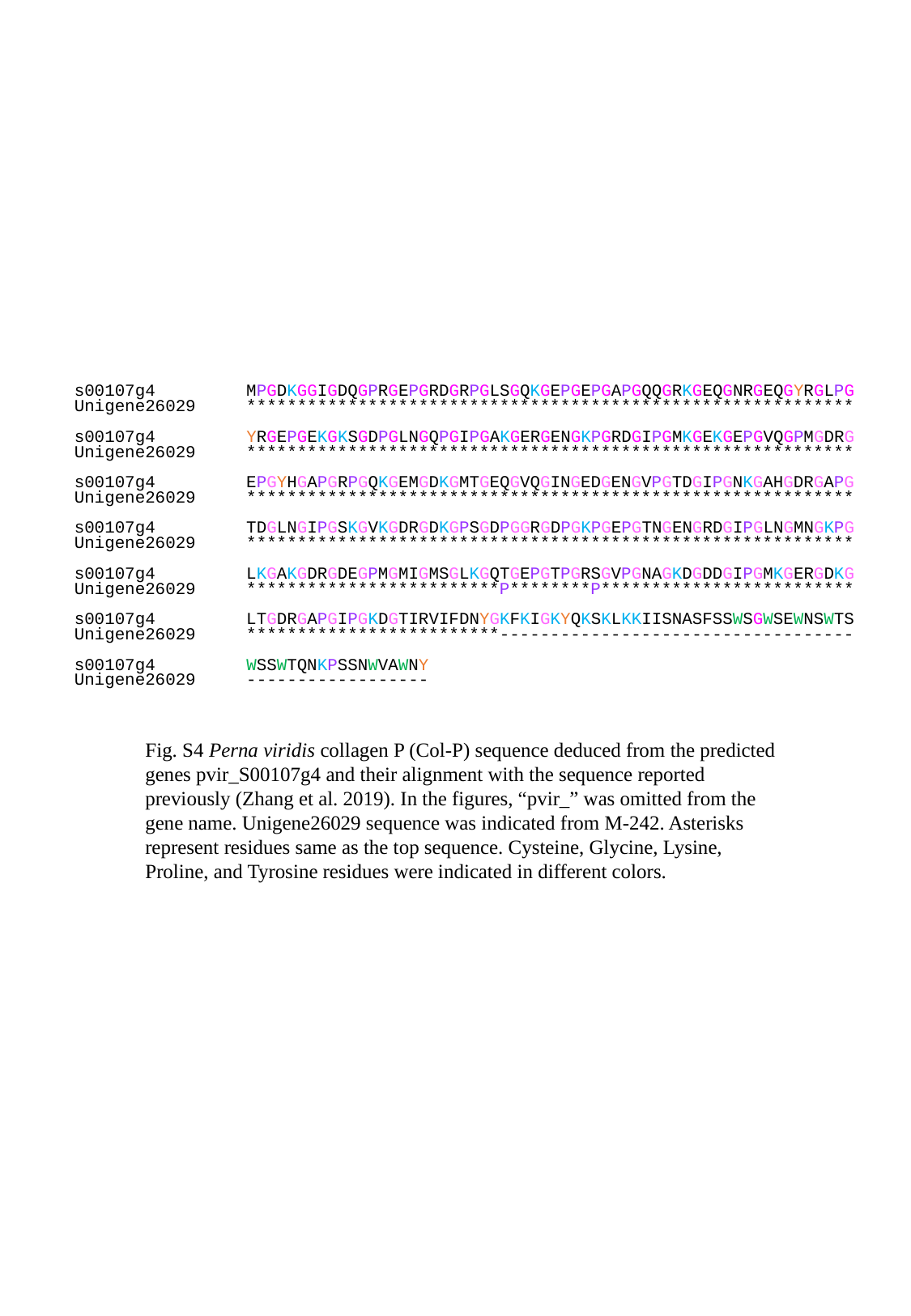

s00107g4 	 MPGDKGGIGDQGPRGEPGRDGRPGLSGQKGEPGEPGAPGQQGRKGEQGNRGEQGYRGLPG
Unigene26029 ************************************************************
s00107g4 	 YRGEPGEKGKSGDPGLNGQPGIPGAKGERGENGKPGRDGIPGMKGEKGEPGVQGPMGDRG
Unigene26029 ************************************************************
s00107g4 	 EPGYHGAPGRPGQKGEMGDKGMTGEQGVQGINGEDGENGVPGTDGIPGNKGAHGDRGAPG
Unigene26029 ************************************************************
s00107g4 	 TDGLNGIPGSKGVKGDRGDKGPSGDPGGRGDPGKPGEPGTNGENGRDGIPGLNGMNGKPG
Unigene26029 ************************************************************
s00107g4 	 LKGAKGDRGDEGPMGMIGMSGLKGQTGEPGTPGRSGVPGNAGKDGDDGIPGMKGERGDKG
Unigene26029 *************************P********P*************************
s00107g4 	 LTGDRGAPGIPGKDGTIRVIFDNYGKFKIGKYQKSKLKKIISNASFSSWSGWSEWNSWTS
Unigene26029 *************************-----------------------------------
s00107g4 	 WSSWTQNKPSSNWVAWNY
Unigene26029 ------------------
Fig. S4 Perna viridis collagen P (Col-P) sequence deduced from the predicted genes pvir_S00107g4 and their alignment with the sequence reported previously (Zhang et al. 2019). In the figures, “pvir_” was omitted from the gene name. Unigene26029 sequence was indicated from M-242. Asterisks represent residues same as the top sequence. Cysteine, Glycine, Lysine, Proline, and Tyrosine residues were indicated in different colors.

## Slide 6
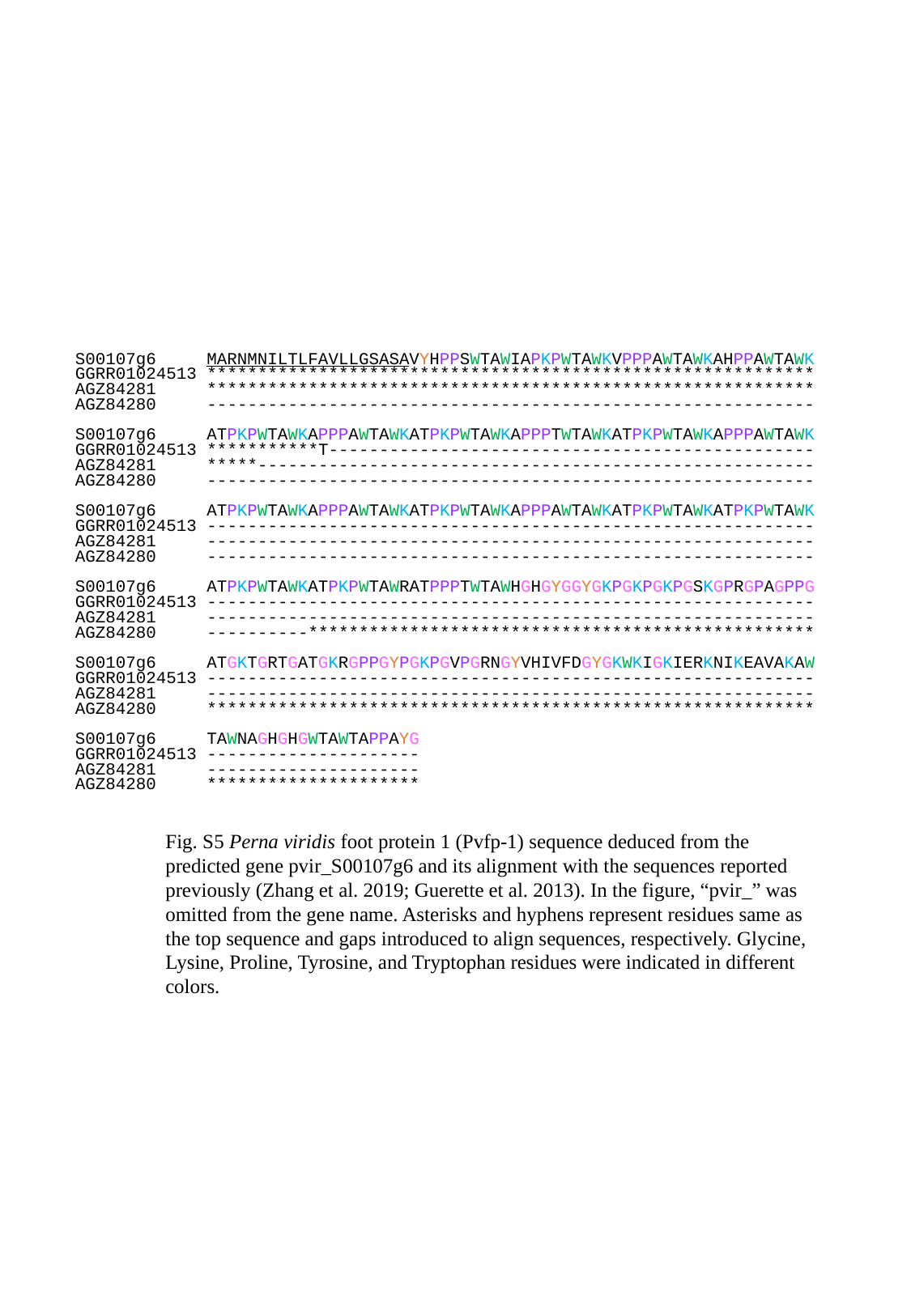

S00107g6 MARNMNILTLFAVLLGSASAVYHPPSWTAWIAPKPWTAWKVPPPAWTAWKAHPPAWTAWK
GGRR01024513 ************************************************************
AGZ84281 ************************************************************
AGZ84280 ------------------------------------------------------------
S00107g6 ATPKPWTAWKAPPPAWTAWKATPKPWTAWKAPPPTWTAWKATPKPWTAWKAPPPAWTAWK
GGRR01024513 ***********T------------------------------------------------
AGZ84281 *****-------------------------------------------------------
AGZ84280 ------------------------------------------------------------
S00107g6 ATPKPWTAWKAPPPAWTAWKATPKPWTAWKAPPPAWTAWKATPKPWTAWKATPKPWTAWK
GGRR01024513 ------------------------------------------------------------
AGZ84281 ------------------------------------------------------------
AGZ84280 ------------------------------------------------------------
S00107g6 ATPKPWTAWKATPKPWTAWRATPPPTWTAWHGHGYGGYGKPGKPGKPGSKGPRGPAGPPG
GGRR01024513 ------------------------------------------------------------
AGZ84281 ------------------------------------------------------------
AGZ84280 ----------**************************************************
S00107g6 ATGKTGRTGATGKRGPPGYPGKPGVPGRNGYVHIVFDGYGKWKIGKIERKNIKEAVAKAW
GGRR01024513 ------------------------------------------------------------
AGZ84281 ------------------------------------------------------------
AGZ84280 ************************************************************
S00107g6 TAWNAGHGHGWTAWTAPPAYG
GGRR01024513 ---------------------
AGZ84281 ---------------------
AGZ84280 *********************
Fig. S5 Perna viridis foot protein 1 (Pvfp-1) sequence deduced from the predicted gene pvir_S00107g6 and its alignment with the sequences reported previously (Zhang et al. 2019; Guerette et al. 2013). In the figure, “pvir_” was omitted from the gene name. Asterisks and hyphens represent residues same as the top sequence and gaps introduced to align sequences, respectively. Glycine, Lysine, Proline, Tyrosine, and Tryptophan residues were indicated in different colors.

## Slide 7
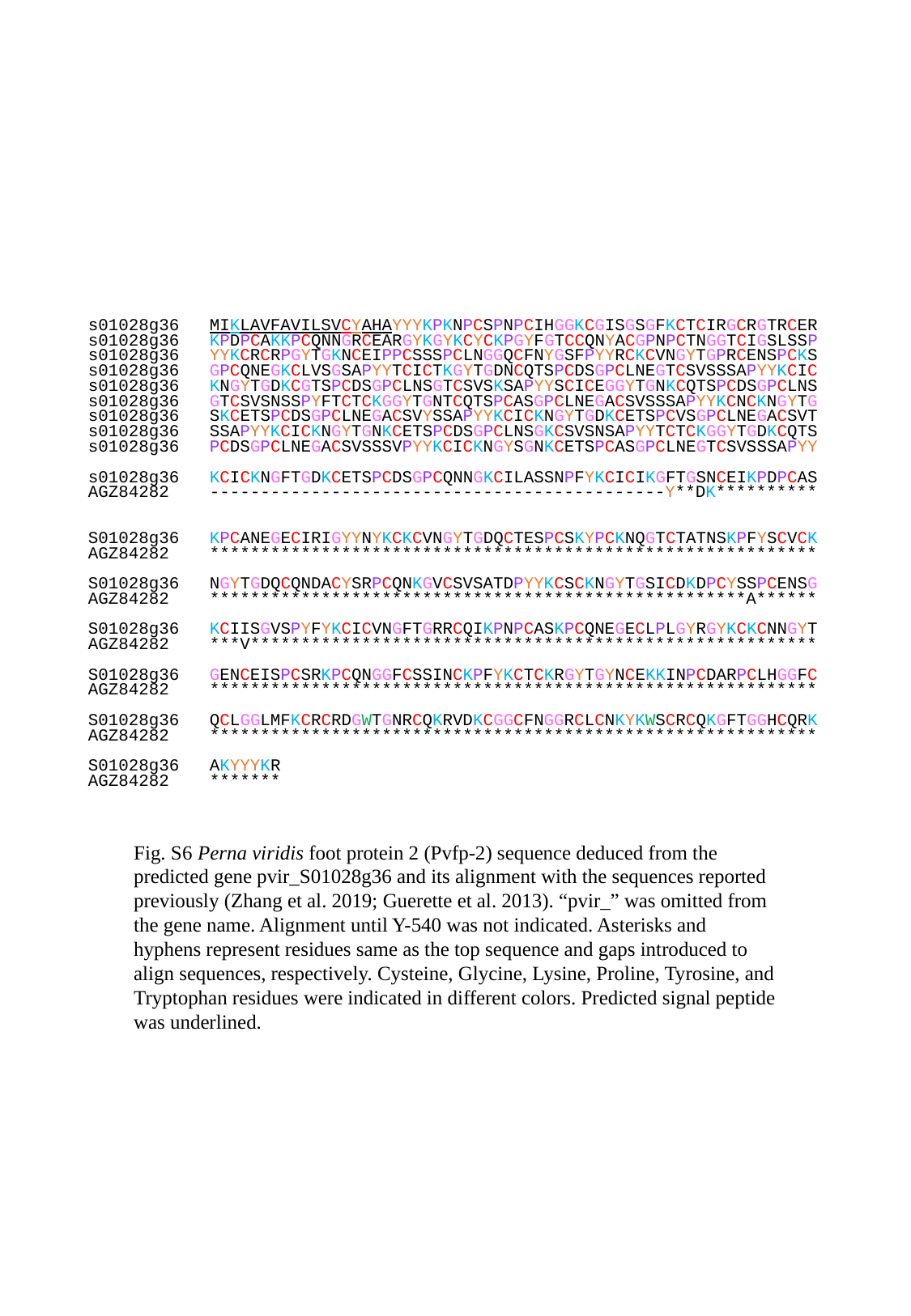

s01028g36	MIKLAVFAVILSVCYAHAYYYKPKNPCSPNPCIHGGKCGISGSGFKCTCIRGCRGTRCER
s01028g36	KPDPCAKKPCQNNGRCEARGYKGYKCYCKPGYFGTCCQNYACGPNPCTNGGTCIGSLSSP
s01028g36	YYKCRCRPGYTGKNCEIPPCSSSPCLNGGQCFNYGSFPYYRCKCVNGYTGPRCENSPCKS
s01028g36	GPCQNEGKCLVSGSAPYYTCICTKGYTGDNCQTSPCDSGPCLNEGTCSVSSSAPYYKCIC
s01028g36	KNGYTGDKCGTSPCDSGPCLNSGTCSVSKSAPYYSCICEGGYTGNKCQTSPCDSGPCLNS
s01028g36	GTCSVSNSSPYFTCTCKGGYTGNTCQTSPCASGPCLNEGACSVSSSAPYYKCNCKNGYTG
s01028g36	SKCETSPCDSGPCLNEGACSVYSSAPYYKCICKNGYTGDKCETSPCVSGPCLNEGACSVT
s01028g36	SSAPYYKCICKNGYTGNKCETSPCDSGPCLNSGKCSVSNSAPYYTCTCKGGYTGDKCQTS
s01028g36	PCDSGPCLNEGACSVSSSVPYYKCICKNGYSGNKCETSPCASGPCLNEGTCSVSSSAPYY
s01028g36	KCICKNGFTGDKCETSPCDSGPCQNNGKCILASSNPFYKCICIKGFTGSNCEIKPDPCAS
AGZ84282 ---------------------------------------------Y**DK**********
S01028g36	KPCANEGECIRIGYYNYKCKCVNGYTGDQCTESPCSKYPCKNQGTCTATNSKPFYSCVCK
AGZ84282	************************************************************
S01028g36	NGYTGDQCQNDACYSRPCQNKGVCSVSATDPYYKCSCKNGYTGSICDKDPCYSSPCENSG
AGZ84282 	*****************************************************A******
S01028g36	KCIISGVSPYFYKCICVNGFTGRRCQIKPNPCASKPCQNEGECLPLGYRGYKCKCNNGYT
AGZ84282 	***V********************************************************
S01028g36	GENCEISPCSRKPCQNGGFCSSINCKPFYKCTCKRGYTGYNCEKKINPCDARPCLHGGFC
AGZ84282 	************************************************************
S01028g36 	QCLGGLMFKCRCRDGWTGNRCQKRVDKCGGCFNGGRCLCNKYKWSCRCQKGFTGGHCQRK
AGZ84282 	************************************************************
S01028g36	AKYYYKR
AGZ84282 	*******
Fig. S6 Perna viridis foot protein 2 (Pvfp-2) sequence deduced from the predicted gene pvir_S01028g36 and its alignment with the sequences reported previously (Zhang et al. 2019; Guerette et al. 2013). “pvir_” was omitted from the gene name. Alignment until Y-540 was not indicated. Asterisks and hyphens represent residues same as the top sequence and gaps introduced to align sequences, respectively. Cysteine, Glycine, Lysine, Proline, Tyrosine, and Tryptophan residues were indicated in different colors. Predicted signal peptide was underlined.

## Slide 8
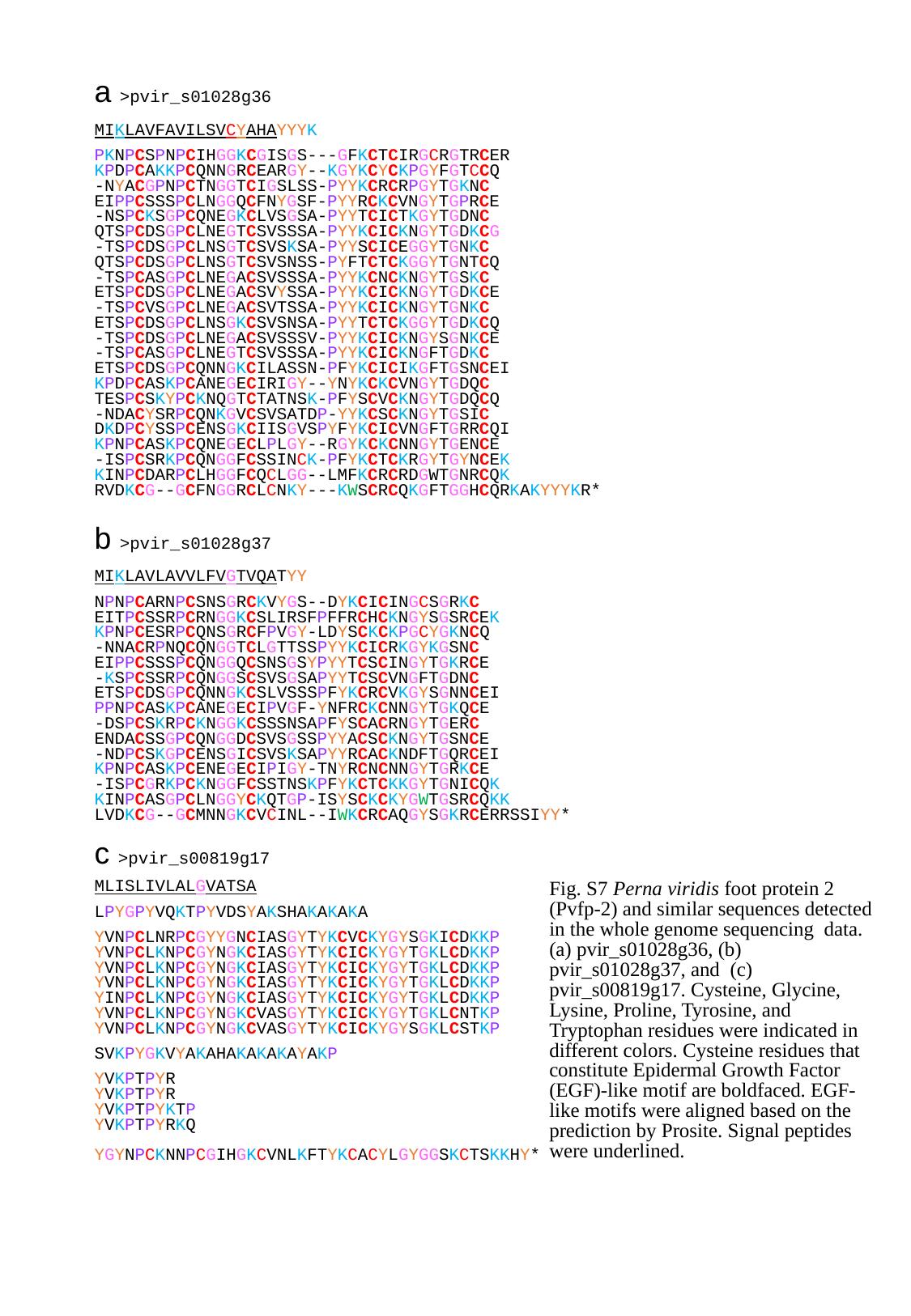

a >pvir_s01028g36
MIKLAVFAVILSVCYAHAYYYK
PKNPCSPNPCIHGGKCGISGS---GFKCTCIRGCRGTRCER
KPDPCAKKPCQNNGRCEARGY--KGYKCYCKPGYFGTCCQ
-NYACGPNPCTNGGTCIGSLSS-PYYKCRCRPGYTGKNC
EIPPCSSSPCLNGGQCFNYGSF-PYYRCKCVNGYTGPRCE
-NSPCKSGPCQNEGKCLVSGSA-PYYTCICTKGYTGDNC
QTSPCDSGPCLNEGTCSVSSSA-PYYKCICKNGYTGDKCG
-TSPCDSGPCLNSGTCSVSKSA-PYYSCICEGGYTGNKC
QTSPCDSGPCLNSGTCSVSNSS-PYFTCTCKGGYTGNTCQ
-TSPCASGPCLNEGACSVSSSA-PYYKCNCKNGYTGSKC
ETSPCDSGPCLNEGACSVYSSA-PYYKCICKNGYTGDKCE
-TSPCVSGPCLNEGACSVTSSA-PYYKCICKNGYTGNKC
ETSPCDSGPCLNSGKCSVSNSA-PYYTCTCKGGYTGDKCQ
-TSPCDSGPCLNEGACSVSSSV-PYYKCICKNGYSGNKCE
-TSPCASGPCLNEGTCSVSSSA-PYYKCICKNGFTGDKC
ETSPCDSGPCQNNGKCILASSN-PFYKCICIKGFTGSNCEI
KPDPCASKPCANEGECIRIGY--YNYKCKCVNGYTGDQC
TESPCSKYPCKNQGTCTATNSK-PFYSCVCKNGYTGDQCQ
-NDACYSRPCQNKGVCSVSATDP-YYKCSCKNGYTGSIC
DKDPCYSSPCENSGKCIISGVSPYFYKCICVNGFTGRRCQI
KPNPCASKPCQNEGECLPLGY--RGYKCKCNNGYTGENCE
-ISPCSRKPCQNGGFCSSINCK-PFYKCTCKRGYTGYNCEK
KINPCDARPCLHGGFCQCLGG--LMFKCRCRDGWTGNRCQK
RVDKCG--GCFNGGRCLCNKY---KWSCRCQKGFTGGHCQRKAKYYYKR*
b >pvir_s01028g37
MIKLAVLAVVLFVGTVQATYY
NPNPCARNPCSNSGRCKVYGS--DYKCICINGCSGRKC
EITPCSSRPCRNGGKCSLIRSFPFFRCHCKNGYSGSRCEK
KPNPCESRPCQNSGRCFPVGY-LDYSCKCKPGCYGKNCQ
-NNACRPNQCQNGGTCLGTTSSPYYKCICRKGYKGSNC
EIPPCSSSPCQNGGQCSNSGSYPYYTCSCINGYTGKRCE
-KSPCSSRPCQNGGSCSVSGSAPYYTCSCVNGFTGDNC
ETSPCDSGPCQNNGKCSLVSSSPFYKCRCVKGYSGNNCEI
PPNPCASKPCANEGECIPVGF-YNFRCKCNNGYTGKQCE
-DSPCSKRPCKNGGKCSSSNSAPFYSCACRNGYTGERC
ENDACSSGPCQNGGDCSVSGSSPYYACSCKNGYTGSNCE
-NDPCSKGPCENSGICSVSKSAPYYRCACKNDFTGQRCEI
KPNPCASKPCENEGECIPIGY-TNYRCNCNNGYTGRKCE
-ISPCGRKPCKNGGFCSSTNSKPFYKCTCKKGYTGNICQK
KINPCASGPCLNGGYCKQTGP-ISYSCKCKYGWTGSRCQKK
LVDKCG--GCMNNGKCVCINL--IWKCRCAQGYSGKRCERRSSIYY*
c >pvir_s00819g17
MLISLIVLALGVATSA
LPYGPYVQKTPYVDSYAKSHAKAKAKA
YVNPCLNRPCGYYGNCIASGYTYKCVCKYGYSGKICDKKP
YVNPCLKNPCGYNGKCIASGYTYKCICKYGYTGKLCDKKP
YVNPCLKNPCGYNGKCIASGYTYKCICKYGYTGKLCDKKP
YVNPCLKNPCGYNGKCIASGYTYKCICKYGYTGKLCDKKP
YINPCLKNPCGYNGKCIASGYTYKCICKYGYTGKLCDKKP
YVNPCLKNPCGYNGKCVASGYTYKCICKYGYTGKLCNTKP
YVNPCLKNPCGYNGKCVASGYTYKCICKYGYSGKLCSTKP
SVKPYGKVYAKAHAKAKAKAYAKP
YVKPTPYR
YVKPTPYR
YVKPTPYKTP
YVKPTPYRKQ
YGYNPCKNNPCGIHGKCVNLKFTYKCACYLGYGGSKCTSKKHY*
Fig. S7 Perna viridis foot protein 2 (Pvfp-2) and similar sequences detected in the whole genome sequencing data. (a) pvir_s01028g36, (b) pvir_s01028g37, and (c) pvir_s00819g17. Cysteine, Glycine, Lysine, Proline, Tyrosine, and Tryptophan residues were indicated in different colors. Cysteine residues that constitute Epidermal Growth Factor (EGF)-like motif are boldfaced. EGF-like motifs were aligned based on the prediction by Prosite. Signal peptides were underlined.

## Slide 9
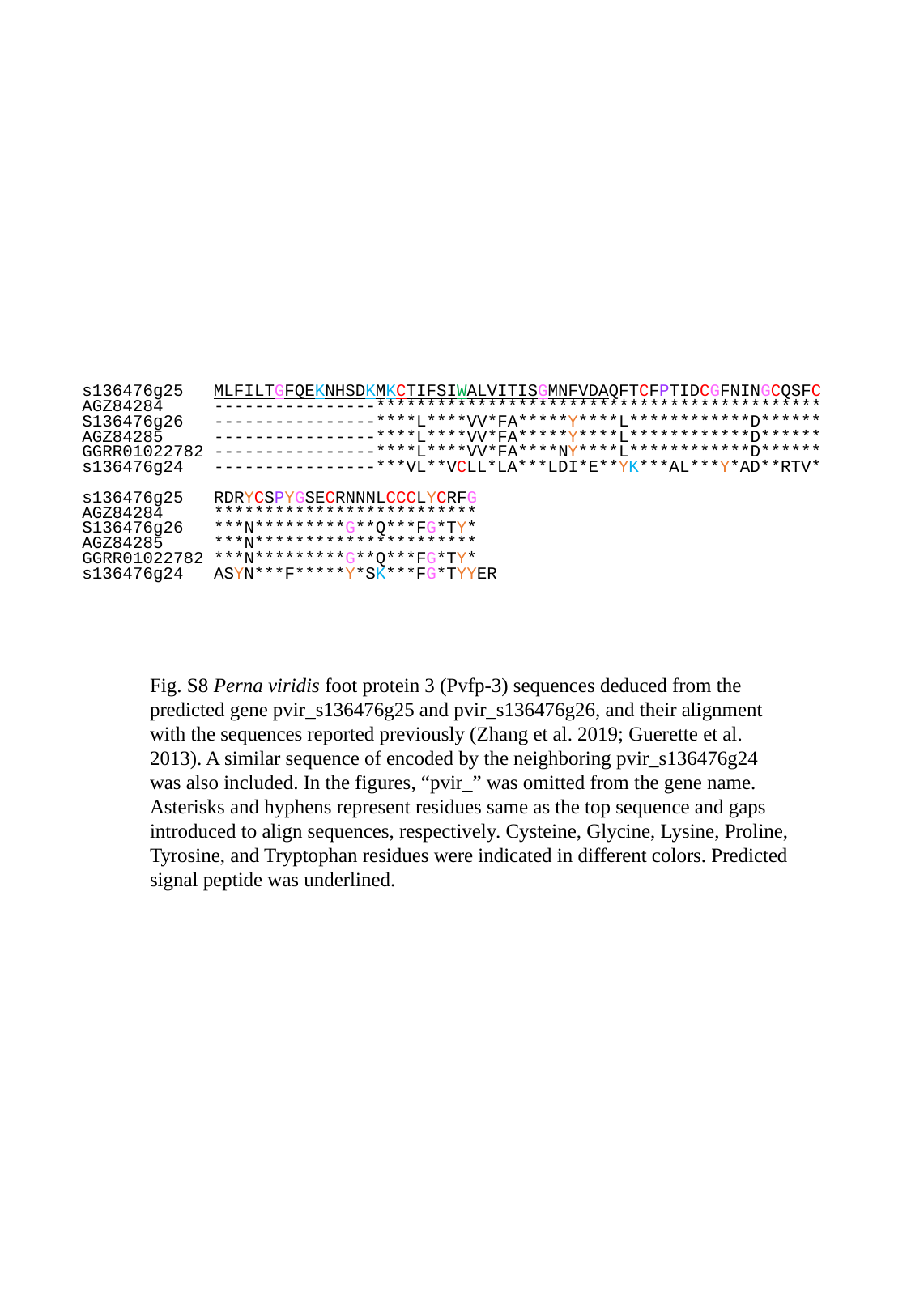

s136476g25 MLFILTGFQEKNHSDKMKCTIFSIWALVITISGMNFVDAQFTCFPTIDCGFNINGCQSFC
AGZ84284 ----------------********************************************
S136476g26 ----------------****L****VV*FA*****Y****L************D******
AGZ84285 ----------------****L****VV*FA*****Y****L************D******
GGRR01022782 ----------------****L****VV*FA****NY****L************D******
s136476g24 ----------------***VL**VCLL*LA***LDI*E**YK***AL***Y*AD**RTV*
s136476g25 RDRYCSPYGSECRNNNLCCCLYCRFG
AGZ84284 **************************
S136476g26 ***N*********G**Q***FG*TY*
AGZ84285 ***N**********************
GGRR01022782 ***N*********G**Q***FG*TY*
s136476g24 ASYN***F*****Y*SK***FG*TYYER
Fig. S8 Perna viridis foot protein 3 (Pvfp-3) sequences deduced from the predicted gene pvir_s136476g25 and pvir_s136476g26, and their alignment with the sequences reported previously (Zhang et al. 2019; Guerette et al. 2013). A similar sequence of encoded by the neighboring pvir_s136476g24 was also included. In the figures, “pvir_” was omitted from the gene name. Asterisks and hyphens represent residues same as the top sequence and gaps introduced to align sequences, respectively. Cysteine, Glycine, Lysine, Proline, Tyrosine, and Tryptophan residues were indicated in different colors. Predicted signal peptide was underlined.

## Slide 10
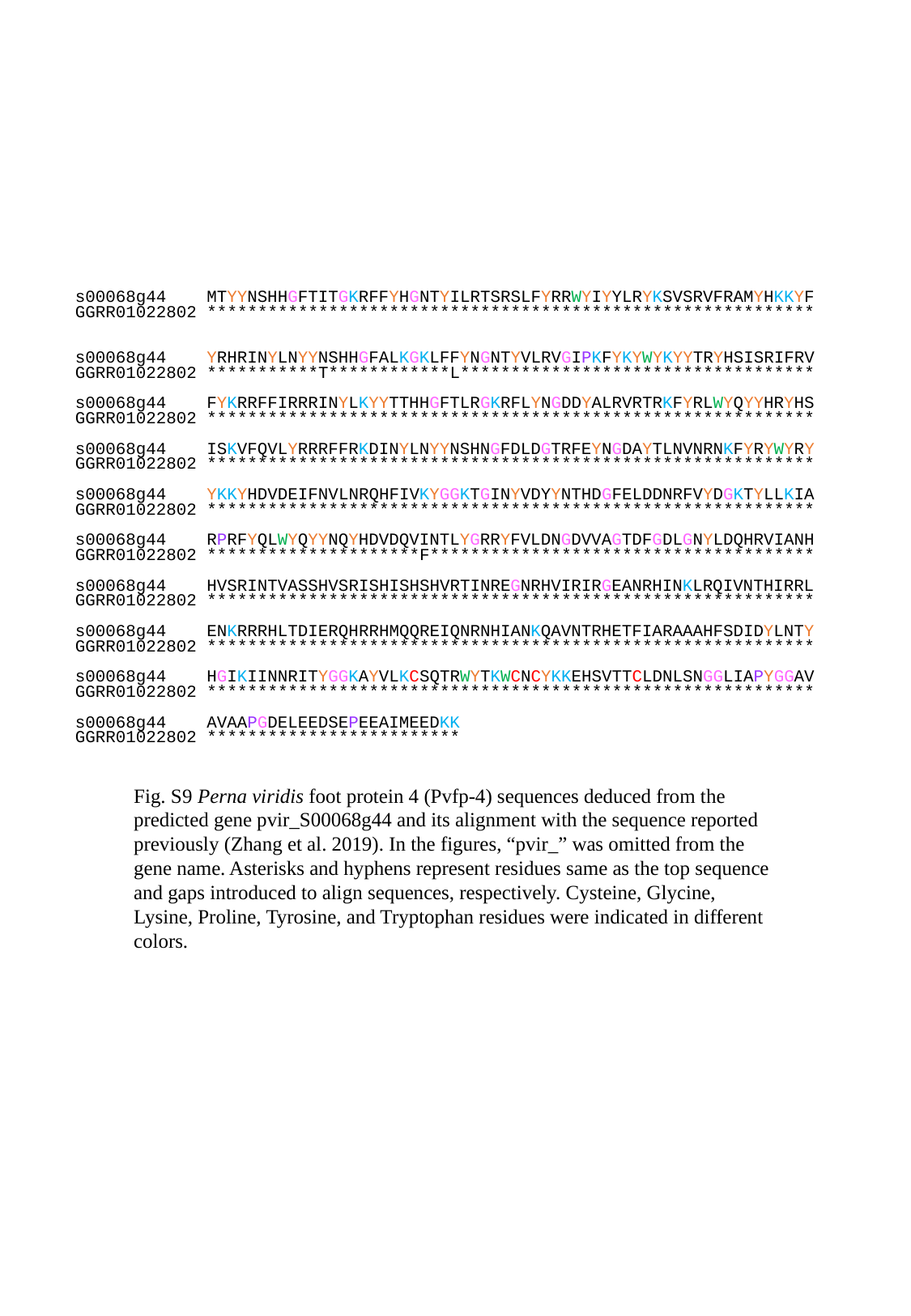

s00068g44 MTYYNSHHGFTITGKRFFYHGNTYILRTSRSLFYRRWYIYYLRYKSVSRVFRAMYHKKYF GGRR01022802 ************************************************************
s00068g44 	 YRHRINYLNYYNSHHGFALKGKLFFYNGNTYVLRVGIPKFYKYWYKYYTRYHSISRIFRV
GGRR01022802 ***********T************L***********************************
s00068g44 	 FYKRRFFIRRRINYLKYYTTHHGFTLRGKRFLYNGDDYALRVRTRKFYRLWYQYYHRYHS GGRR01022802 ************************************************************
s00068g44 	 ISKVFQVLYRRRFFRKDINYLNYYNSHNGFDLDGTRFEYNGDAYTLNVNRNKFYRYWYRY
GGRR01022802 ************************************************************
s00068g44 	 YKKYHDVDEIFNVLNRQHFIVKYGGKTGINYVDYYNTHDGFELDDNRFVYDGKTYLLKIA
GGRR01022802 ************************************************************
s00068g44 	 RPRFYQLWYQYYNQYHDVDQVINTLYGRRYFVLDNGDVVAGTDFGDLGNYLDQHRVIANH
GGRR01022802 *********************F**************************************
s00068g44 	 HVSRINTVASSHVSRISHISHSHVRTINREGNRHVIRIRGEANRHINKLRQIVNTHIRRL
GGRR01022802 ************************************************************
s00068g44 	 ENKRRRHLTDIERQHRRHMQQREIQNRNHIANKQAVNTRHETFIARAAAHFSDIDYLNTY
GGRR01022802 ************************************************************
s00068g44 	 HGIKIINNRITYGGKAYVLKCSQTRWYTKWCNCYKKEHSVTTCLDNLSNGGLIAPYGGAV
GGRR01022802 ************************************************************
s00068g44 	 AVAAPGDELEEDSEPEEAIMEEDKK
GGRR01022802 *************************
Fig. S9 Perna viridis foot protein 4 (Pvfp-4) sequences deduced from the predicted gene pvir_S00068g44 and its alignment with the sequence reported previously (Zhang et al. 2019). In the figures, “pvir_” was omitted from the gene name. Asterisks and hyphens represent residues same as the top sequence and gaps introduced to align sequences, respectively. Cysteine, Glycine, Lysine, Proline, Tyrosine, and Tryptophan residues were indicated in different colors.

## Slide 11
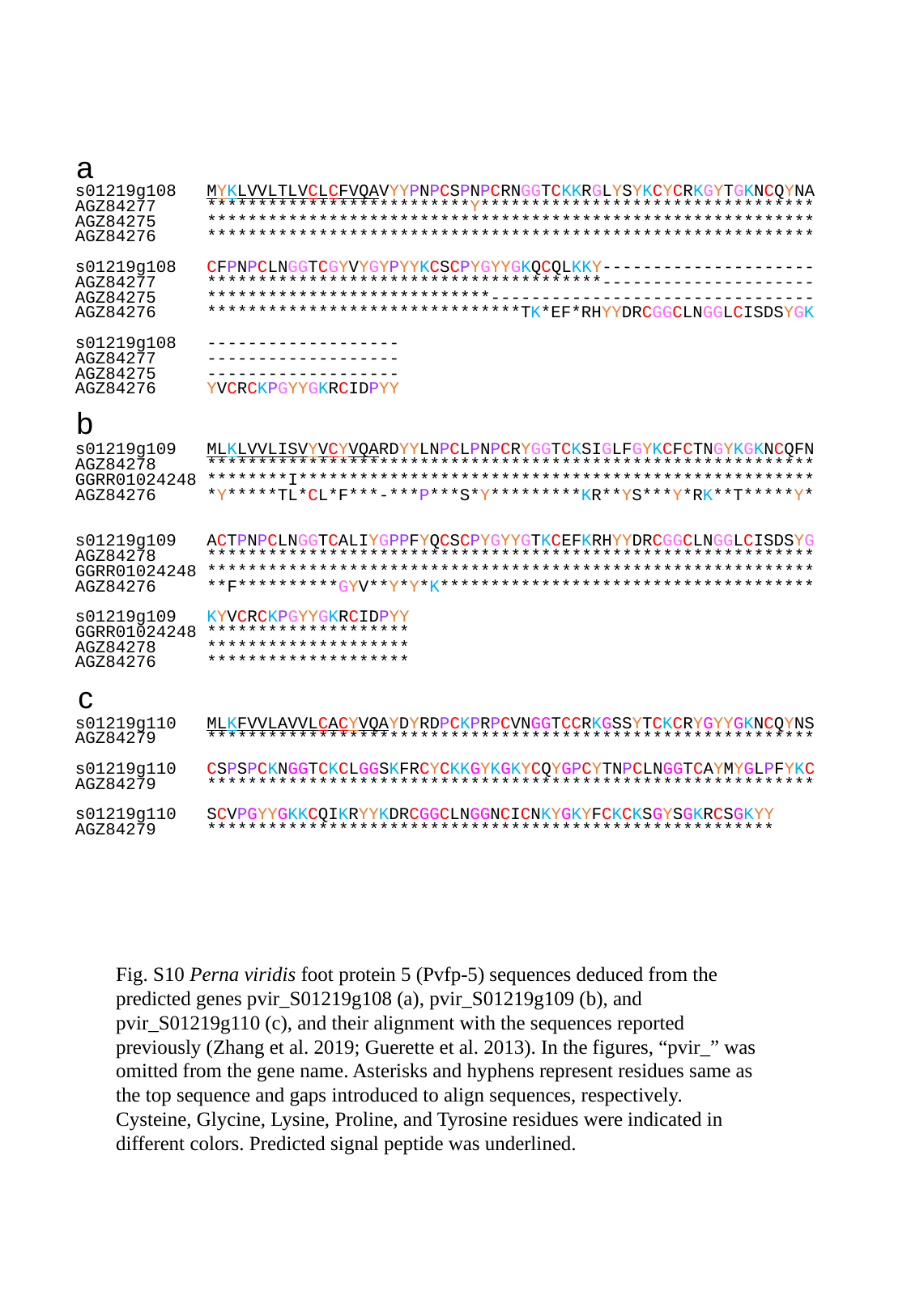

a
s01219g108 MYKLVVLTLVCLCFVQAVYYPNPCSPNPCRNGGTCKKRGLYSYKCYCRKGYTGKNCQYNA
AGZ84277 **************************Y*********************************
AGZ84275 ************************************************************
AGZ84276 ************************************************************
s01219g108 CFPNPCLNGGTCGYVYGYPYYKCSCPYGYYGKQCQLKKY---------------------
AGZ84277 ***************************************---------------------
AGZ84275 ****************************--------------------------------
AGZ84276 *******************************TK*EF*RHYYDRCGGCLNGGLCISDSYGK
s01219g108 -------------------
AGZ84277 -------------------
AGZ84275 -------------------
AGZ84276 YVCRCKPGYYGKRCIDPYY
s01219g109 MLKLVVLISVYVCYVQARDYYLNPCLPNPCRYGGTCKSIGLFGYKCFCTNGYKGKNCQFN
AGZ84278 ************************************************************
GGRR01024248 ********I***************************************************
AGZ84276 *Y*****TL*CL*F***-***P***S*Y*********KR**YS***Y*RK**T*****Y*
s01219g109 ACTPNPCLNGGTCALIYGPPFYQCSCPYGYYGTKCEFKRHYYDRCGGCLNGGLCISDSYG
AGZ84278 ************************************************************
GGRR01024248 ************************************************************
AGZ84276 **F**********GYV**Y*Y*K*************************************
s01219g109 KYVCRCKPGYYGKRCIDPYY
GGRR01024248 ********************
AGZ84278 ********************
AGZ84276 ********************
s01219g110 MLKFVVLAVVLCACYVQAYDYRDPCKPRPCVNGGTCCRKGSSYTCKCRYGYYGKNCQYNS
AGZ84279 ************************************************************
s01219g110 CSPSPCKNGGTCKCLGGSKFRCYCKKGYKGKYCQYGPCYTNPCLNGGTCAYMYGLPFYKC
AGZ84279 ************************************************************
s01219g110 SCVPGYYGKKCQIKRYYKDRCGGCLNGGNCICNKYGKYFCKCKSGYSGKRCSGKYY
AGZ84279 ********************************************************
b
c
Fig. S10 Perna viridis foot protein 5 (Pvfp-5) sequences deduced from the predicted genes pvir_S01219g108 (a), pvir_S01219g109 (b), and pvir_S01219g110 (c), and their alignment with the sequences reported previously (Zhang et al. 2019; Guerette et al. 2013). In the figures, “pvir_” was omitted from the gene name. Asterisks and hyphens represent residues same as the top sequence and gaps introduced to align sequences, respectively. Cysteine, Glycine, Lysine, Proline, and Tyrosine residues were indicated in different colors. Predicted signal peptide was underlined.

## Slide 12
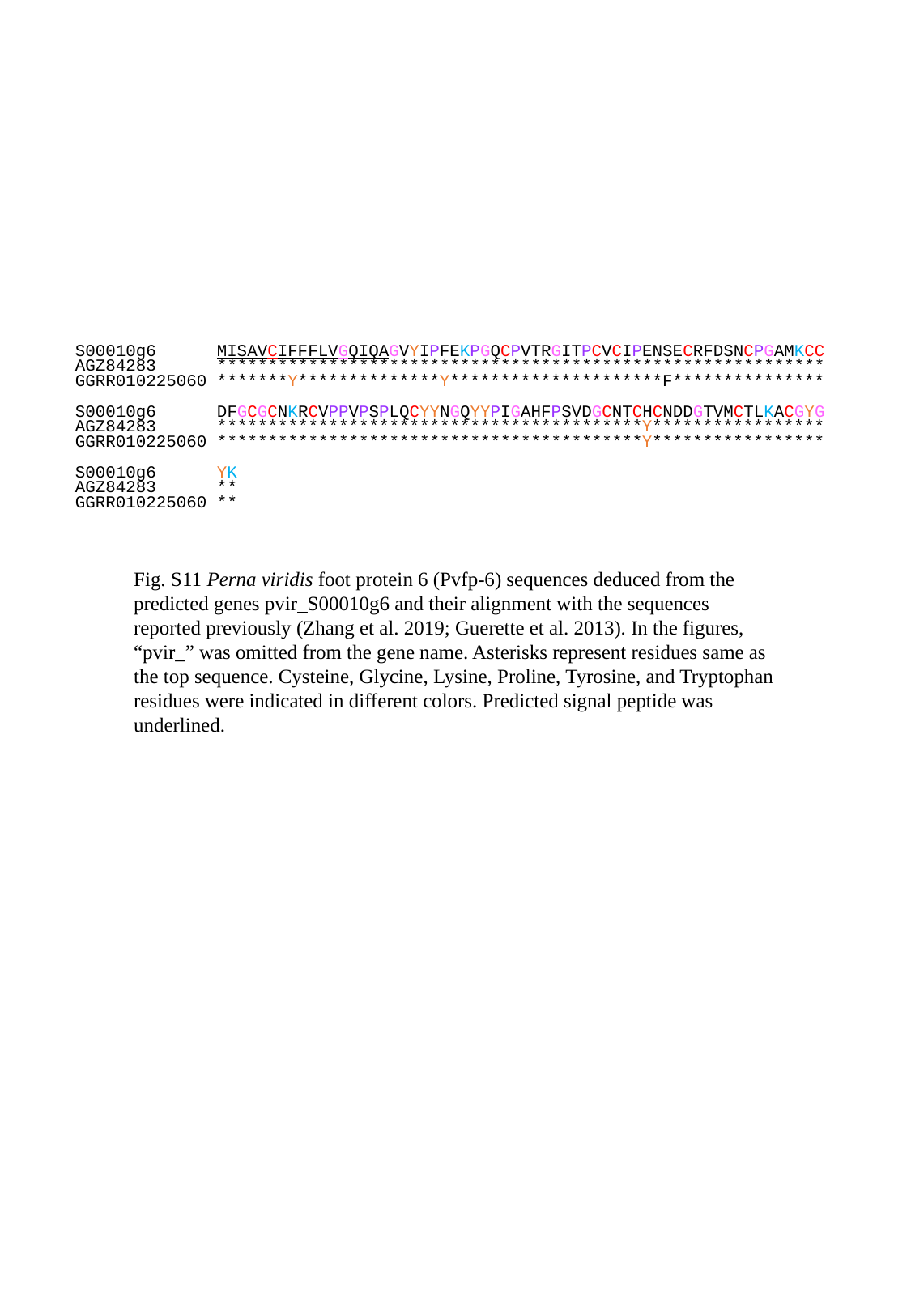

S00010g6 	 MISAVCIFFFLVGQIQAGVYIPFEKPGQCPVTRGITPCVCIPENSECRFDSNCPGAMKCC
AGZ84283 ************************************************************
GGRR010225060 *******Y**************Y*********************F***************
S00010g6 DFGCGCNKRCVPPVPSPLQCYYNGQYYPIGAHFPSVDGCNTCHCNDDGTVMCTLKACGYG
AGZ84283 ******************************************Y*****************
GGRR010225060 ******************************************Y*****************
S00010g6 YK
AGZ84283 **
GGRR010225060 **
Fig. S11 Perna viridis foot protein 6 (Pvfp-6) sequences deduced from the predicted genes pvir_S00010g6 and their alignment with the sequences reported previously (Zhang et al. 2019; Guerette et al. 2013). In the figures, “pvir_” was omitted from the gene name. Asterisks represent residues same as the top sequence. Cysteine, Glycine, Lysine, Proline, Tyrosine, and Tryptophan residues were indicated in different colors. Predicted signal peptide was underlined.

## Slide 13
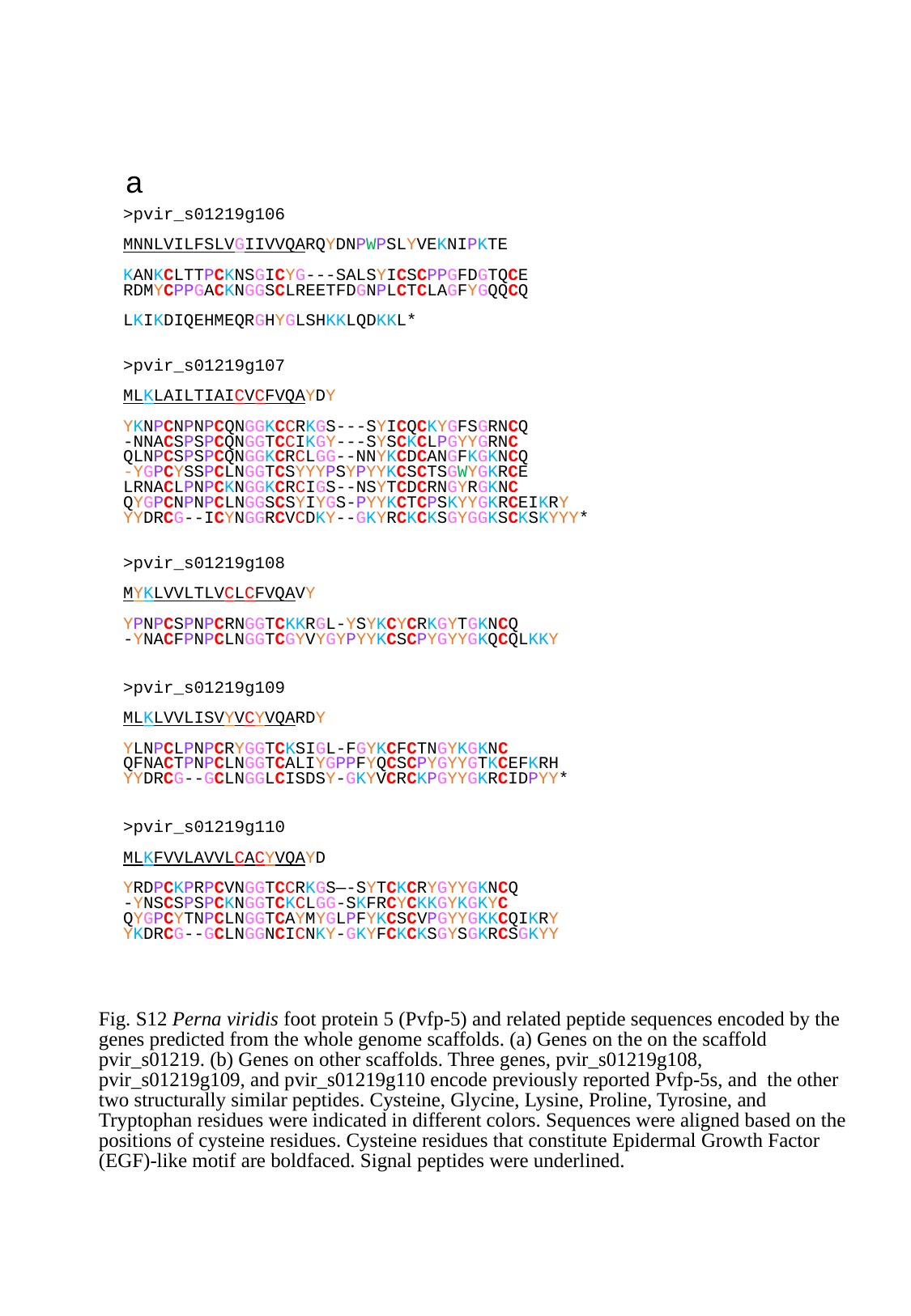

a
>pvir_s01219g106
MNNLVILFSLVGIIVVQARQYDNPWPSLYVEKNIPKTE
KANKCLTTPCKNSGICYG---SALSYICSCPPGFDGTQCE
RDMYCPPGACKNGGSCLREETFDGNPLCTCLAGFYGQQCQ
LKIKDIQEHMEQRGHYGLSHKKLQDKKL*
>pvir_s01219g107
MLKLAILTIAICVCFVQAYDY
YKNPCNPNPCQNGGKCCRKGS---SYICQCKYGFSGRNCQ
-NNACSPSPCQNGGTCCIKGY---SYSCKCLPGYYGRNC
QLNPCSPSPCQNGGKCRCLGG--NNYKCDCANGFKGKNCQ
-YGPCYSSPCLNGGTCSYYYPSYPYYKCSCTSGWYGKRCE
LRNACLPNPCKNGGKCRCIGS--NSYTCDCRNGYRGKNC
QYGPCNPNPCLNGGSCSYIYGS-PYYKCTCPSKYYGKRCEIKRY
YYDRCG--ICYNGGRCVCDKY--GKYRCKCKSGYGGKSCKSKYYY*
>pvir_s01219g108
MYKLVVLTLVCLCFVQAVY
YPNPCSPNPCRNGGTCKKRGL-YSYKCYCRKGYTGKNCQ
-YNACFPNPCLNGGTCGYVYGYPYYKCSCPYGYYGKQCQLKKY
>pvir_s01219g109
MLKLVVLISVYVCYVQARDY
YLNPCLPNPCRYGGTCKSIGL-FGYKCFCTNGYKGKNC
QFNACTPNPCLNGGTCALIYGPPFYQCSCPYGYYGTKCEFKRH
YYDRCG--GCLNGGLCISDSY-GKYVCRCKPGYYGKRCIDPYY*
>pvir_s01219g110
MLKFVVLAVVLCACYVQAYD
YRDPCKPRPCVNGGTCCRKGS—-SYTCKCRYGYYGKNCQ
-YNSCSPSPCKNGGTCKCLGG-SKFRCYCKKGYKGKYC
QYGPCYTNPCLNGGTCAYMYGLPFYKCSCVPGYYGKKCQIKRY
YKDRCG--GCLNGGNCICNKY-GKYFCKCKSGYSGKRCSGKYY
Fig. S12 Perna viridis foot protein 5 (Pvfp-5) and related peptide sequences encoded by the genes predicted from the whole genome scaffolds. (a) Genes on the on the scaffold pvir_s01219. (b) Genes on other scaffolds. Three genes, pvir_s01219g108, pvir_s01219g109, and pvir_s01219g110 encode previously reported Pvfp-5s, and the other two structurally similar peptides. Cysteine, Glycine, Lysine, Proline, Tyrosine, and Tryptophan residues were indicated in different colors. Sequences were aligned based on the positions of cysteine residues. Cysteine residues that constitute Epidermal Growth Factor (EGF)-like motif are boldfaced. Signal peptides were underlined.

## Slide 14
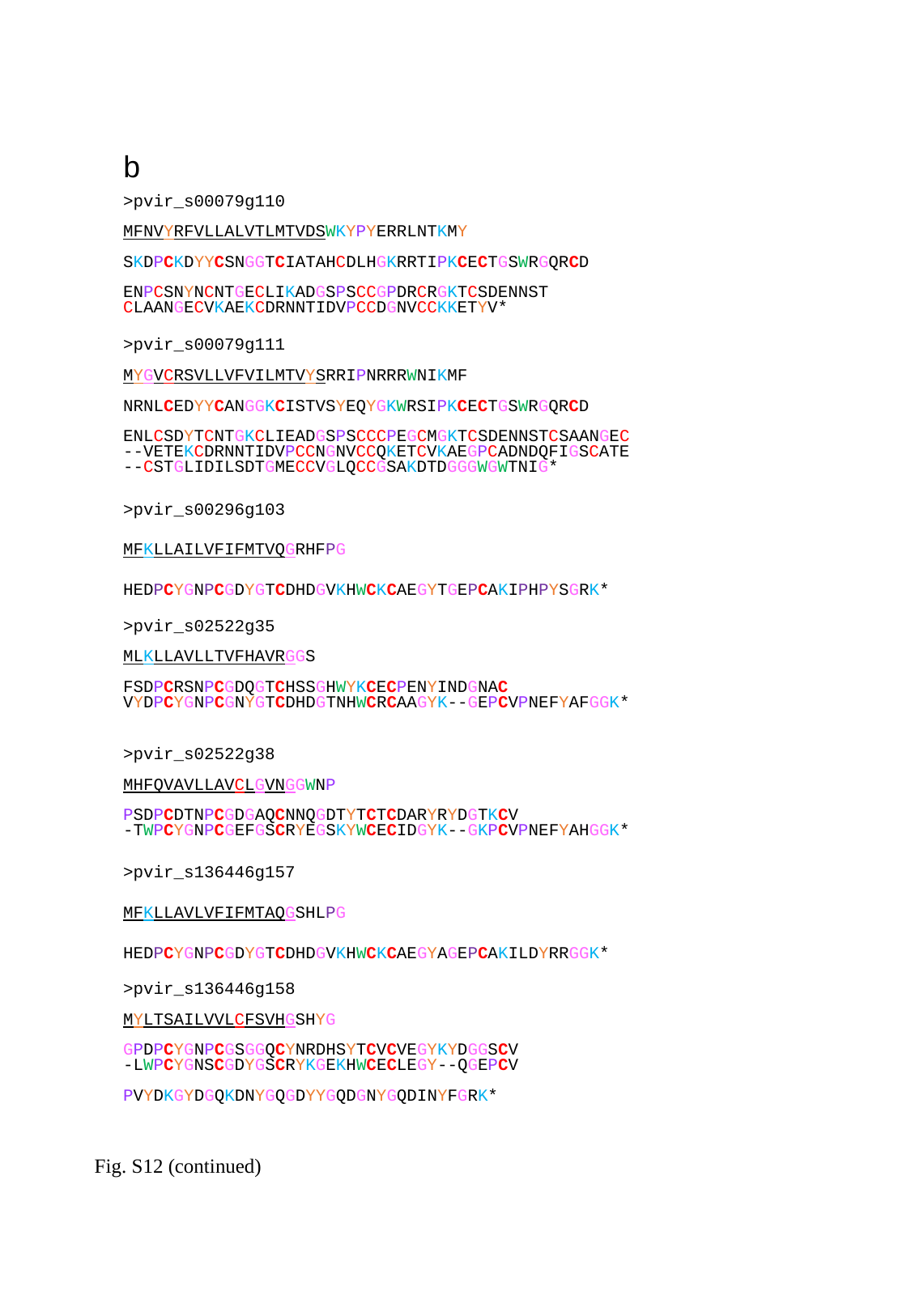

b
>pvir_s00079g110
MFNVYRFVLLALVTLMTVDSWKYPYERRLNTKMY
SKDPCKDYYCSNGGTCIATAHCDLHGKRRTIPKCECTGSWRGQRCD
ENPCSNYNCNTGECLIKADGSPSCCGPDRCRGKTCSDENNST
CLAANGECVKAEKCDRNNTIDVPCCDGNVCCKKETYV*
>pvir_s00079g111
MYGVCRSVLLVFVILMTVYSRRIPNRRRWNIKMF
NRNLCEDYYCANGGKCISTVSYEQYGKWRSIPKCECTGSWRGQRCD
ENLCSDYTCNTGKCLIEADGSPSCCCPEGCMGKTCSDENNSTCSAANGEC
--VETEKCDRNNTIDVPCCNGNVCCQKETCVKAEGPCADNDQFIGSCATE
--CSTGLIDILSDTGMECCVGLQCCGSAKDTDGGGWGWTNIG*
>pvir_s00296g103
MFKLLAILVFIFMTVQGRHFPG
HEDPCYGNPCGDYGTCDHDGVKHWCKCAEGYTGEPCAKIPHPYSGRK*
>pvir_s02522g35
MLKLLAVLLTVFHAVRGGS
FSDPCRSNPCGDQGTCHSSGHWYKCECPENYINDGNAC
VYDPCYGNPCGNYGTCDHDGTNHWCRCAAGYK--GEPCVPNEFYAFGGK*
>pvir_s02522g38
MHFQVAVLLAVCLGVNGGWNP
PSDPCDTNPCGDGAQCNNQGDTYTCTCDARYRYDGTKCV
-TWPCYGNPCGEFGSCRYEGSKYWCECIDGYK--GKPCVPNEFYAHGGK*
>pvir_s136446g157
MFKLLAVLVFIFMTAQGSHLPG
HEDPCYGNPCGDYGTCDHDGVKHWCKCAEGYAGEPCAKILDYRRGGK*
>pvir_s136446g158
MYLTSAILVVLCFSVHGSHYG
GPDPCYGNPCGSGGQCYNRDHSYTCVCVEGYKYDGGSCV
-LWPCYGNSCGDYGSCRYKGEKHWCECLEGY--QGEPCV
PVYDKGYDGQKDNYGQGDYYGQDGNYGQDINYFGRK*
Fig. S12 (continued)

## Slide 15
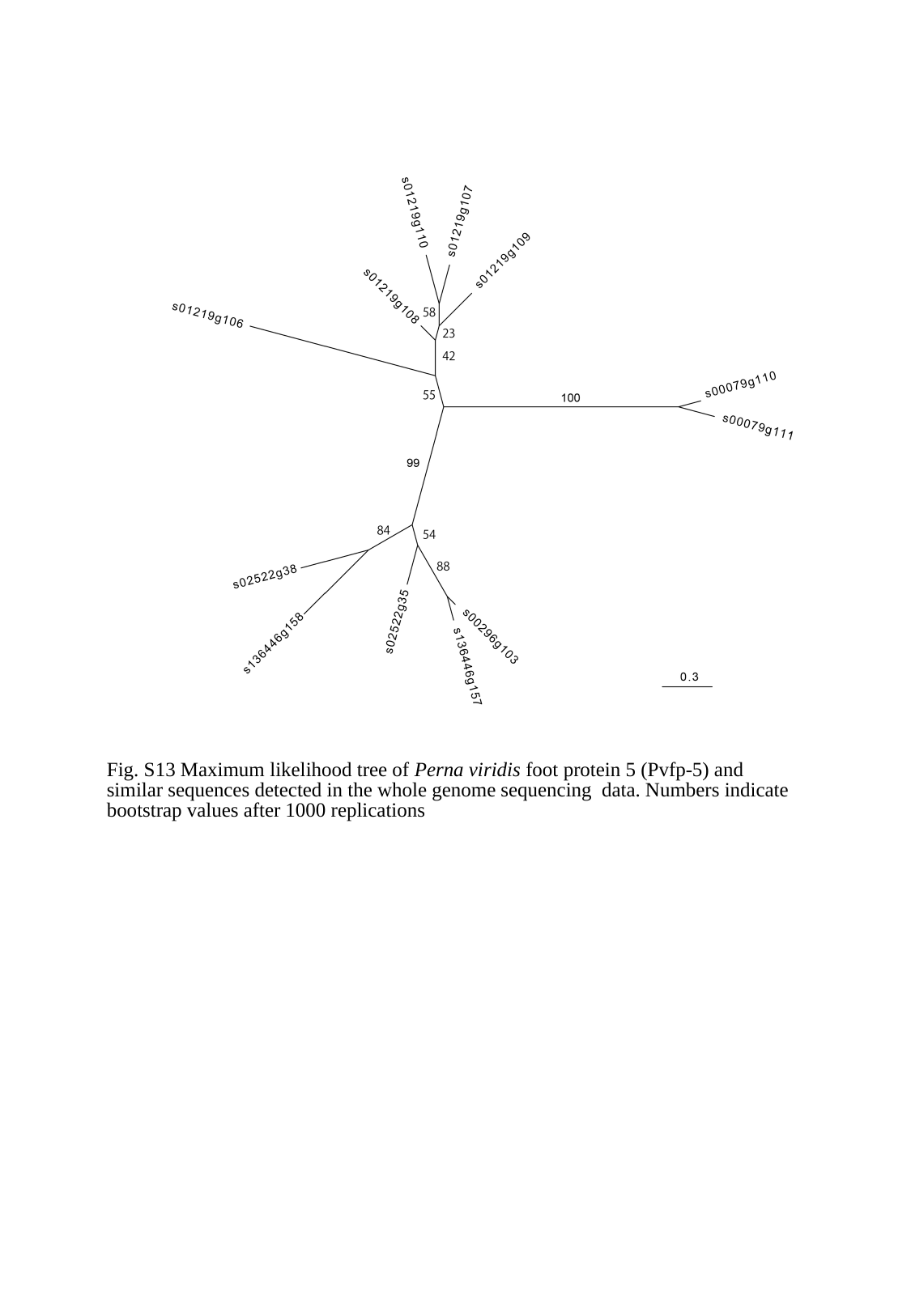

Fig. S13 Maximum likelihood tree of Perna viridis foot protein 5 (Pvfp-5) and similar sequences detected in the whole genome sequencing data. Numbers indicate bootstrap values after 1000 replications
