# Supplementary material for: Genomics and Transcriptomics of the green mussel explain the durability of its byssus
Source: Sci Rep. 2021 Mar 16;11:5992. doi: 10.1038/s41598-021-84948-6 (PMC7971044; doi:10.1038/s41598-021-84948-6)
Supplement: Supplementary file 2 — Supplementary Table S1. [file 41598_2021_84948_MOESM2_ESM.docx]

**Supplementary Table S1**

For

**Genomics of the green mussel explain the durability of its byssus**

Koji Inoue, Yuki Yoshioka, Hiroyuki Tanaka, Azusa Kinjo, Mieko Sassa, Ikuo Ueda, Chuya Shinzato, Atsushi Toyoda, Takehiko Itoh

Table S1 Sequence libraries used for whole genome sequencing of the green mussel *Perna viridis.*

| Library | Read Length | Pair number | Nucleotide number | Nucleotide number after trimming |
| --- | --- | --- | --- | --- |
| PE600 | 250 bp | 120M | 60.0 Gb | 57.8 Gb |
| MP3k | 100 bp | 30M | 8.0 Gb | 5.3 Gb |
| MP6k | 100 bp | 30M | 8.1 Gb | 5.3 Gb |
| MP10k | 100 bp | 30M | 8.8 Gb | 5.3 Gb |
| MP15k | 100 bp | 29M | 7.9 Gb | 5.0 Gb |
