# Supplementary material for: Genomics and Transcriptomics of the green mussel explain the durability of its byssus
Source: Sci Rep. 2021 Mar 16;11:5992. doi: 10.1038/s41598-021-84948-6 (PMC7971044; doi:10.1038/s41598-021-84948-6)
Supplement: Supplementary file 3 — Supplementary Information Table S2. [file 41598_2021_84948_MOESM3_ESM.pdf]

Supplementary Table S2

For

Genomics of the green mussel explain the durability of its byssus

Koji Inoue, Yuki Yoshioka, Hiroyuki Tanaka, Azusa Kinjo, Mieko Sassa, Ikuo Ueda, Chuya Shinzato, Atsushi Toyoda, Takehiko Itoh

Table S2 Domains found amplified in *Perna viridis* genome compared to bivalve and gastropod species.

Motif ID	Name	***Perna viridis***	*Bathymodiolus platifrons*	*Modiolus philippinarum*	*Limnoperna fortunei*	*Pinctada fucata*	*Crassostrea gigas*	*Patinopecten yessoensis*	*Haliotis discus hannai*	*Lottia gigantea*
PF00059	Lectin C-type domain	295	185	289	212	239	222	163	109	111
PF00095	WAP-type (Whey Acidic Protein) 'four-disulfide core'	41	25	20	21	8	15	11	4	15
PF00193	Extracellular link domain	11	0	2	1	0	1	0	0	1
PF00782	Dual specificity phosphatase, catalytic domain	60	38	36	38	40	47	39	16	40
PF08659	KR domain	92	52	74	51	69	62	87	26	63
PF13350	Tyrosine phosphatase family	18	2	4	3	4	5	6	2	1
