# Supplementary material for: Genomics and Transcriptomics of the green mussel explain the durability of its byssus
Source: Sci Rep. 2021 Mar 16;11:5992. doi: 10.1038/s41598-021-84948-6 (PMC7971044; doi:10.1038/s41598-021-84948-6)
Supplement: Supplementary file 4 — Supplementary Information Table S3. [file 41598_2021_84948_MOESM4_ESM.docx]

**Supplementary Table S3**

For

**Whole-genome and transcriptome analyses of the green mussel reveal a hidden strategy to make the byssus durable**

Koji Inoue, Yuki Yoshioka, Hiroyuki Tanaka, Azusa Kinjo, Mieko Sassa, Ikuo Ueda, Chuya Shinzato, Atsushi Toyoda, Takehiko Itoh

Table S2 Summary of the data used for phylogenetic analysis
